# The performance status gap in immunotherapy for frail patients with advanced non-small cell lung cancer

**DOI:** 10.1007/s00262-024-03763-w

**Published:** 2024-07-02

**Authors:** Julie Tsu-Yu Wu, June Corrigan, Chloe Su, Clark Dumontier, Jennifer La, Aparjita Khan, Shipra Arya, Alex H. S. Harris, Leah Backhus, Millie Das, Nhan V. Do, Mary T. Brophy, Summer S. Han, Michael Kelley, Nathanael R. Fillmore

**Affiliations:** 1 VA Palo Alto Healthcare System, Stanford University, Palo Alto, CA USA; 2 VA Boston Healthcare System, Boston, USA; 3 Stanford University, Palo Alto, CA USA; 4 VA Boston Healthcare System, Harvard Medical School, Boston, USA; 5 VA Boston Healthcare System, Boston University School of Medicine, Boston, USA; 6 Durham VA Healthcare System, Duke University, Durham, NC USA; 7 VA Boston Healthcare System, Dana-Farber Cancer Institute, Harvard Medical School, Boston, USA; 8 Massachusetts Veterans Epidemiology Research and Information Center, 150 S Huntington Ave, Boston, MA 02141 USA

**Keywords:** Frailty, Performance status, Lung cancer

## Abstract

**Purpose:**

In advanced non-small cell lung cancer (NSCLC), immune checkpoint inhibitor (ICI) monotherapy is often preferred over intensive ICI treatment for frail patients and those with poor performance status (PS). Among those with poor PS, the additional effect of frailty on treatment selection and mortality is unknown.

**Methods:**

Patients in the veterans affairs national precision oncology program from 1/2019–12/2021 who received first-line ICI for advanced NSCLC were followed until death or study end 6/2022. Association of an electronic frailty index with treatment selection was examined using logistic regression stratified by PS. We also examined overall survival (OS) on intensive treatment using Cox regression stratified by PS. Intensive treatment was defined as concurrent use of platinum-doublet chemotherapy and/or dual checkpoint blockade and non-intensive as ICI monotherapy.

**Results:**

Of 1547 patients receiving any ICI, 66.2% were frail, 33.8% had poor PS (≥ 2), and 25.8% were both. Frail patients received less intensive treatment than non-frail patients in both PS subgroups (Good PS: odds ratio [OR] 0.67, 95% confidence interval [CI] 0.51 − 0.88; Poor PS: OR 0.69, 95% CI 0.44 − 1.10). Among 731 patients receiving intensive treatment, frailty was associated with lower OS for those with good PS (hazard ratio [HR] 1.53, 95% CI 1.2 − 1.96), but no association was observed with poor PS (HR 1.03, 95% CI 0.67 − 1.58).

**Conclusion:**

Frail patients with both good and poor PS received less intensive treatment. However, frailty has a limited effect on survival among those with poor PS. These findings suggest that PS, not frailty, drives survival on intensive treatment.

**Supplementary Information:**

The online version contains supplementary material available at 10.1007/s00262-024-03763-w.

## Introduction

Lung cancer is the leading cause of cancer-related mortality [1] and is common among older patients [2], who are frequently more frail than younger patients [3, 4]. Frail patients have reduced physiological reserve and increased vulnerability to stressors like cancer [5–7]. Frailty often encompasses functional domains but also includes nutritional, cognitive, physical and social domains of frailty [8, 9]. It is distinct from performance status (PS), a well-established measure of functional status defined by patients’ ability to carry out daily activities and self-care [10]. Frailty, as measured by an electronic index, refers to a pre-existing state before cancer [5, 11]. However, patients with advanced non-small cell lung cancer (NSCLC) commonly present with acute, cancer-related symptoms that rapidly decrease their function and therefore their PS score [12, 13].

Oncologists often adjust treatment intensity for frailty and poor PS based on intuitive assessments made in clinic, favoring immune checkpoint inhibitor (ICI) monotherapy instead of more intensive options like chemoimmunotherapy in advanced NSCLC. Frail patients may be unable to receive intense chemotherapy due to impaired kidney or liver function, leading to a lower rate of response and shorter treatment duration [14, 15]. They may also face a higher risk of treatment-related complications that require early cessation of treatment [16, 17]. Patients with poor PS face similar risks of increased toxicity, which may worsen outcomes [18]. However, the risks for patients with both frailty and poor PS, frequently observed in those with advanced NSCLC [12, 19], are unknown. I’s uncertain if poor PS would worsen treatment toxicity due to frailty, potentially negatively impacting survival, or if the significant negative prognostic effects of poor PS would outweigh any additional negative impact from frailty.

Despite their importance in clinical decisions, the interaction between frailty and PS and its impact on treatment decisions and outcomes are not well understood. One key challenge is the limited data availability of these two factors. Clinical trials have focused on healthy patients with good PS [20, 21], and cancer registries do not capture frailty nor PS at the time of advanced disease [22]. Accordingly, most prior NSCLC population-based studies did not evaluate the combined impact of frailty and PS [13, 18, 23–26]. To enable analyses for this study, we employed natural language processing (NLP) and an electronic frailty index to efficiently extract PS and frailty, respectively, from the veterans affairs (VA) electronic medical record (EMR) [27, 28].

Our study aimed to investigate the association between frailty and treatment selection within PS strata in a nationwide, multi-institutional cohort of patients receiving first-line ICI treatment for advanced NSCLC. We stratified the analysis to compare patients with good (< 2) and poor (≥ 2) PS, considering that concerns about additive toxicity with poor PS may not apply to good PS. We hypothesized that oncologists would prefer ICI monotherapy for frail patients, anticipating worse outcomes on intense treatment than non-frail patients. We expected this preference would remain even when PD-L1 expression predicted a low chance of response to ICI monotherapy. Next, we evaluated whether frail patients exhibited worse survival on intense treatment, thus supporting oncologists’ treatment preferences, and whether frailty’s association with mortality increased or was attenuated among those with poor PS.

## Methods

### Data sources

We conducted an observational cohort study using multi-institutional data from the VA cancer registry; the VA corporate data warehouse, which centralizes EMR data for patients seen at VA facilities nationwide; and the VA national precision oncology program, which provides centralized tumor molecular testing to VA oncology practices across the country [29]. Patient-level data were curated as described in the Supplement. The study was approved by the VA Boston healthcare system institutional review board, and a waiver of informed consent was granted.

### Population

**Fig. 1:**
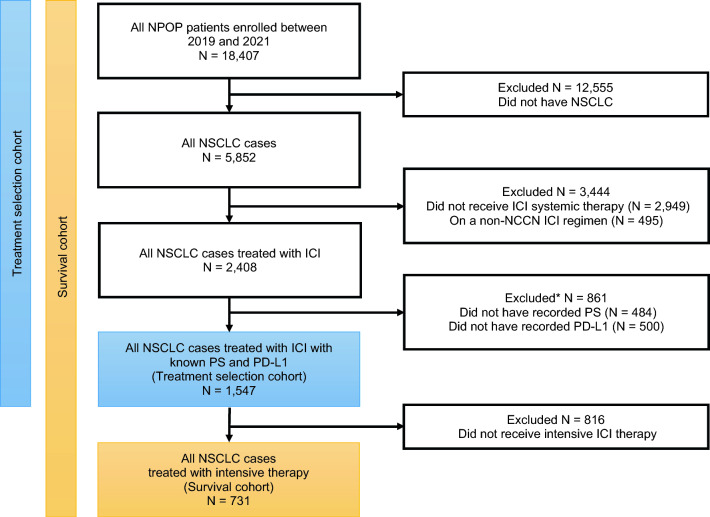
Overview of the study cohort. ICI, immune checkpoint inhibitor; NCCN, national comprehensive cancer network; NPOP, national precision oncology program; NSCLC, non-small cell lung cancer; PD-L1, programmed death ligand 1; PS, performance status; *Patients may have both unknown PS and unknown PD-L1, so the sum of patients with unknown PS and patients with unknown PD-L1 is greater than the total number excluded

We included patients enrolled in the VA National Precision Oncology Program between January 1, 2019, and December 30, 2021, and followed until death or last patient encounter until the end of the study period on June 26, 2022. Patients who were diagnosed with NSCLC and initiated first-line ICI treatment were included (Fig. 1). Patients who did not receive systemic treatment, received first-line agents not listed in the national comprehensive cancer network (NCCN) guidelines for advanced NSCLC [30], or did not have recorded PD-L1 or PS were excluded. To assess treatment selection, the cohort included all patients who received first-line ICI treatment. To assess overall survival (OS) on intensive treatment, the cohort was restricted to the subset of patients who received first-line intensive ICI treatment.

### Study outcome

To first assess treatment selection, the primary outcome was receipt of intensive over non-intensive first-line treatment. Intensive treatment was defined as dual checkpoint blockade and/or chemoimmunotherapy. For dual checkpoint blockade, CTLA-4 inhibitor and PD-1/PD-L1 inhibitor must have been initiated on the same date. For chemoimmunotherapy, chemotherapy must be started within 30 days before to 30 days after ICI initiation date. Chemotherapy was defined as platinum-doublet regimens listed in NCCN guidelines for NSCLC [30]. ICI monotherapy (non-intensive treatment) was defined as first-line ICI without concurrent platinum-doublet regimen or dual checkpoint blockade. To next assess survival on intensive treatment, the primary outcome was OS from the date of treatment initiation to the date of death.

### Exposure and stratification factor

The primary exposure was frailty, measured by our previously validated VA frailty index (VA-FI) [3] as described in the Supplement. Briefly, the VA-FI is defined as the number of age-related health deficits observed in an individual patient, out of 31 possible deficits. The presence of a deficit was defined by receipt of an associated diagnosis or procedure code within the 3 years prior to treatment initiation date. The categories frail (VA-FI > 0.2) or non-frail (≤ 0.2) were set using a previously defined threshold [3, 27].

The primary stratification factor for subgroup analyses was PS. PS at diagnosis was obtained using NLP as described in the Supplement. Briefly, PS was extracted from clinical notes dated from 60 days prior to 14 days after treatment initiation date. PS was classified as good (PS < 2) or poor (≥ 2) based on typical trial eligibility criteria, which predominantly select patients with PS < 2 [20]. If PS was represented as a range (e.g., 1–2), the upper end of the range was used.

### Statistical analysis

*Treatment selection:* Among all patients on first-line ICI, odds ratios (ORs) for the association between frailty and PS with the outcome of receipt of intensive treatment (versus ICI monotherapy) were calculated using multivariate logistic regression. ORs were adjusted for age, gender, race/ethnicity, smoking status at time of initiation of treatment, cancer histology, cancer stage at initial diagnosis, and PD-L1 expression. To assess the effect of frailty on treatment selection in different PS subgroups, we also conducted the analysis on patients stratified by PS (< 2 and ≥ 2).

*OS on intensive treatment:* Among patients treated with intensive treatment, Kaplan–Meier curves and multivariate Cox regression analysis were used to assess the effect of frailty and PS on OS. Cox regression was adjusted for the same covariates as in the treatment selection analysis. Similarly, to assess the effect of frailty on OS in different PS subgroups, we repeated the analyses for patients stratified by PS (< 2 and ≥ 2).

*Missing data handling:* Missing structured data were supplemented by NLP as detailed in the Supplement. Variable missingness after this combined approach is shown in Supplemental Table 1. We conducted our primary analysis with unknown values as a separate category for each covariate. For a sensitivity analysis, we imputed the value of missing data with multiple imputation by chained equations with five imputations, with the assumption that data were missing at random [31]. The imputed data sets were used to assess treatment selection and OS, and estimates from the five imputed data sets were pooled using Rubin’s rules.

*Sensitivity analyses:* To observe PDL1-specific trends, we stratified analyses by PD-L1: negative (0%), low (1–49%), and high (≥ 50%). To examine the similarity between the effects of age and frailty, we divided the cohort based on age (< 65 vs. ≥ 65 years) and utilized age as the exposure variable instead of frailty. To account for potential changes in PS over time, estimates of 0–6 month and ≥ 6 month hazard ratios (HRs) were calculated. We chose 6 months as a timepoint by which there should be treatment response or cancer progression in most patients [32–34], which may change PS. To examine the impact of frailty on OS beyond intensive treatment, we repeated the survival analysis for ICI monotherapy. We also compared OS on intensive treatment versus immunotherapy directly. All statistical analyses were performed using R version 4.0.3.

## Results

### Baseline characteristics

Among the 1547 patients overall, two-thirds were classified as frail (*N* = 1024, 66.2%) and a third had PS 2 or higher (*N* = 523, 33.8%) (Table 1). The most common PS was 1 (PS 0: 362 (23.4%), PS 1: 662 (42.8%), PS 2: 395 (25.5%), PS 3: 116 (7.5%), and PS 4: 12 (0.8%)). Frailty and PS were closely related, with frail patients more likely to have poor PS (≥ 2) and vice versa (Supplemental Tables 2 and 3). Frail patients with poor PS constituted over a quarter of the cohort (*N* = 400, 25.8%).

**Table 1:** Patient characteristics by treatment type

	Type of ICI treatment					
	Total		Non-intensive*		Intensive*	
	N	(%)	N	(%)	N	(%)
Total no. of patients	1547		816		731	
Demographics						
Mean (SD)	70.5	7.43	71.2	7.41	69.8	7.39
*Age strata*						
< 65 years old	339	21.9	168	20.6	171	23.4
65 + years old	1208	78.1	648	79.4	560	76.6
*Sex*						
Male	1496	96.7	787	96.4	709	97.0
Female	51	3.3	29	3.6	22	3.0
*Race*						
Non-hispanic white	1089	70.4	569	69.7	520	71.1
Black	331	21.4	188	23.0	143	19.6
Other/unknown	127	8.2	59	7.2	68	9.3
*Smoking status†*						
Current	806	52.1	416	51.0	390	53.4
Former	649	42.0	362	44.4	287	39.3
Never/unknown	92	5.9	38	4.7	54	7.4
Clinical characteristics§						
*Frailty*						
Not frail	523	33.8	230	28.2	293	40.1
Frail	1024	66.2	586	71.8	438	59.9
*Performance status*						
0–1	1024	66.2	487	59.7	537	73.5
2 or greater	523	33.8	329	40.3	194	26.5
Tumor characteristics						
*Histology*						
Adenocarcinoma	731	47.3	370	45.3	361	49.4
Squamous cell	321	20.7	192	23.5	129	17.6
Other/unknown	495	32.0	254	31.1	241	33.0
*Stage at initial diagnosis*						
I	205	13.3	111	13.6	94	12.9
II	90	5.8	70	8.6	20	2.7
III	227	14.7	160	19.6	67	9.2
IV	690	44.6	310	38.0	380	52.0
Unknown	335	21.7	165	20.2	170	23.3
*PD-L1 expression‡*						
Negative	457	29.5	180	22.1	277	37.9
Low	511	33.0	232	28.4	279	38.2
High	579	37.4	404	49.5	175	23.9

ICI, immune checkpoint inhibitor; PD-L1, programmed death ligand 1; * Intensive ICI treatment was defined as receipt of first-line dual checkpoint blockade or chemoimmunotherapy, as detailed in Methods. Non-intensive therapy was defined as receipt of first-line ICI without dual checkpoint blockade or chemotherapy

^†^ Smoking status is at time of ICI treatment initiation

^§^ Clinical characteristics (frailty and performance status) were measured at the time of ICI treatment initiation

^‡^ PD-L1 expression levels were defined as follows: Negative 0%, Low 1–49%, High 50% or greater

Frailty and PS were associated with distinct patient differences (Supplemental Tables 2 and 3). Compared to non-frail patients, frail patients tended to be older and diagnosed at an earlier stage. PD-L1 expression did not appreciably differ by frailty (Supplemental Table 2). Similarly, patients with poor PS tended to be older than those with good PS. However, unlike frailty, patients with poor PS exhibited higher PD-L1 expression, suggesting that any ICI treatment was primarily offered when PD-L1 expression was high (Supplemental Table 3).

### Treatment selection in the all-treatment cohort

Of the 1547 patients on any first-line immunotherapy, 731 (47.2%) were on intensive treatment and 816 (52.7%) were on ICI monotherapy (Table 1). Overall, patients on intensive treatment were younger, less frail, had lower PD-L1 expression, and were less likely to have poor PS compared to those on ICI monotherapy. The most frequently used intensive treatment regimen was carboplatin, pemetrexed, and pembrolizumab (452 of 731 patients, 61.8%), and the most frequently used non-intensive treatment regimen was pembrolizumab (679 of 816 patients, 83.2%, Supplemental Table 4).

To evaluate whether PS and frailty were independent predictors of treatment selection, we conducted multivariate logistic regression adjusted for covariates, such as PD-L1 (Supplemental Table 5). Independent of PS, frail patients were less likely than non-frail patients to receive intense treatment (OR 0.67, 95% confidence interval [CI] 0.53–0.85, *p* = 9.52 × 10^–4^). Similarly, patients with poor PS were less likely to receive intensive treatment than patients with good PS (OR 0.60, 95% CI 0.48–0.77, *p* = 2.95 × 10^–5^). Sensitivity analysis to estimate missing covariate values through multiple imputation produced consistent results (Supplemental Table 6).

**Fig. 2:**
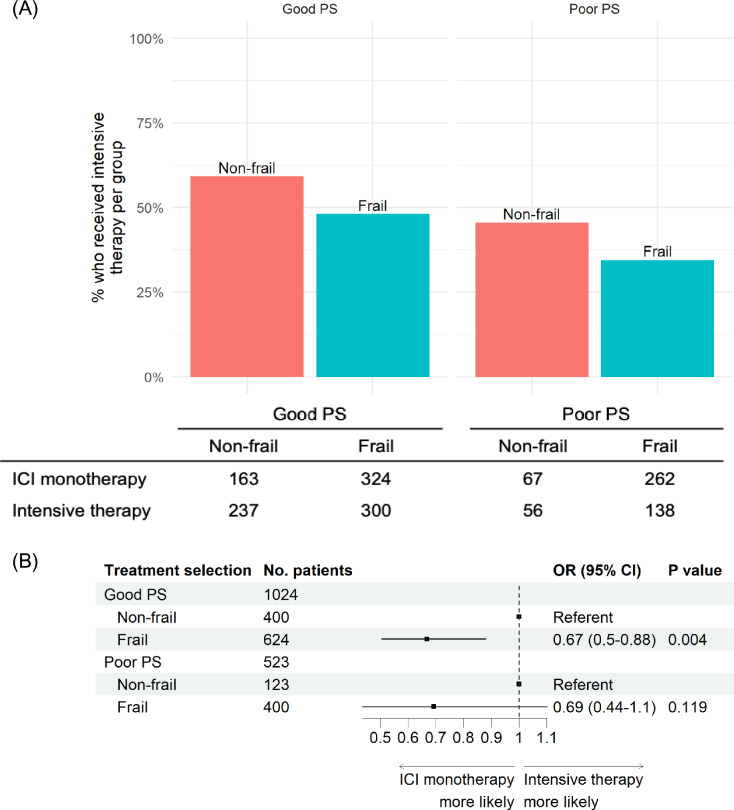
Association of frailty with intensive therapy selection stratified by performance status. **A** First-line immune checkpoint inhibitor (ICI) treatment selection in the all-treatment cohort (*N* = 1547) and stratified by performance status (PS). PS is categorized as good (0–1) or poor (2 or greater) based on clinical notes at time of treatment initiation. Intense therapy is defined as concurrent receipt of platinum-doublet chemotherapy and/or dual checkpoint blockade. Non-intense therapy was defined as receipt of first-line ICI without dual checkpoint blockade or chemotherapy. **B** Forest plot of odds ratio (OR) for treatment selection estimated using multivariable logistic regression adjusting for age, gender, race/ethnicity, smoking status, cancer histology, stage at initial diagnosis, and PD-L1 score. Square symbols indicate the estimates of OR. Error bars indicate the 95% confidence interval (CI)

**Fig. 3:**
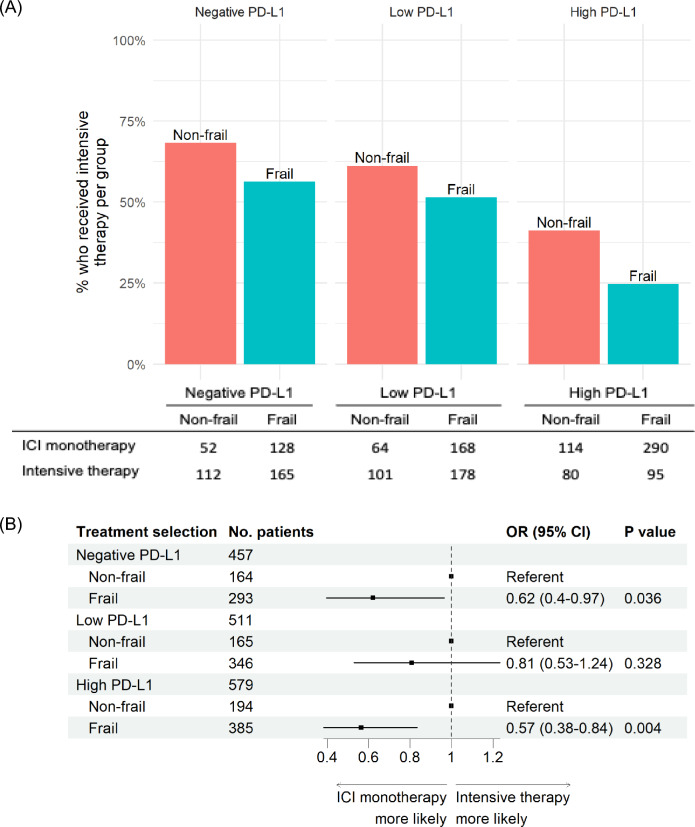
Association of frailty with intensive therapy selection stratified by PD-L1 expression levels. **A** First-line immune checkpoint inhibitor (ICI) treatment selection in the all-treatment cohort (*N* = 1547) and stratified by PD-L1 expression level: negative (0%), low (1–49%), and high (≥ 50%). **B** Forest plot of odds ratio (OR) for treatment selection estimated using multivariable logistic regression adjusting for age, gender, race/ethnicity, smoking status, cancer histology, stage at initial diagnosis, and PS. Square symbols indicate the estimates of OR. Error bars indicate the 95% confidence interval (CI)

To evaluate the effect of frailty in different subgroups, we conducted multivariate logistic regression stratified by PS and PD-L1. Frail patients received less intensive treatment in both PS subgroups (Good PS: OR 0.67, 95% CI 0.51–0.88, *p* = 0.004; Poor PS: OR 0.69, 95% CI 0.44–1.10, *p* = 0.119, Fig. 2). Similarly, across all PD-L1 levels, frail patients preferentially receive ICI monotherapy (Fig. 3). This was observed even when PD-L1 expression was negative and likelihood of response was low. These patients were predominantly frail (128 of 180 patients, 71.1%), although the majority had good PS (129 patients, 71.7%, Supplemental Table 7). Further stratifying by PS, frailty’s association with ICI monotherapy for negative PD-L1 was most evident in the poor PS subgroup (Good PS: OR 0.72, 95% CI 0.43–1.19, *p* = 0.204; Poor PS: OR 0.28, 95% CI 0.09–0.79, *p* = 0.021, Supplemental Fig. 1).

Given the association of age with frailty [3], we examined whether age had a comparable effect on treatment selection as frailty. As expected, patients aged 65 years and older with poor PS tended to receive less intense treatment (Supplemental Fig. 2). However, in contrast to frailty, the association with age was minimal for patients with good PS (Supplemental Fig. 2).

### Overall survival in the intensive treatment cohort

We next examined whether oncologists’ preference for less intense therapy in frail patients aligned with survival outcomes. Specifically, we reasoned that if frailty was associated with poorer survival on intensive treatment, that would support a preference for less intense ICI monotherapy in frail patients.

Among the 731 patients on intensive treatment, median follow-up time was 19.1 months (interquartile range 10.8–28.7 months). During follow-up, there were 410 deaths. PS and frailty significantly impacted OS (Supplemental Fig. 3). Median OS was 13.9 months (95% CI 12.8–16.4 months), compared to 8.7 months (95% CI 7.9–12.5 months) for patients with poor PS and 11.6 months (95% CI 10.2–13.5 months) for frail patients. In our multivariate Cox regression model, both frailty (HR 1.35, 95% CI 1.10–1.68, *p* = 0.005) and PS (HR 1.52, 95% CI 1.22–1.89, *p* = 0.001) were independently associated with OS (Supplemental Table 8). Imputing missing adjustment factor values further strengthened both associations (Supplemental Table 9).

**Fig. 4:**
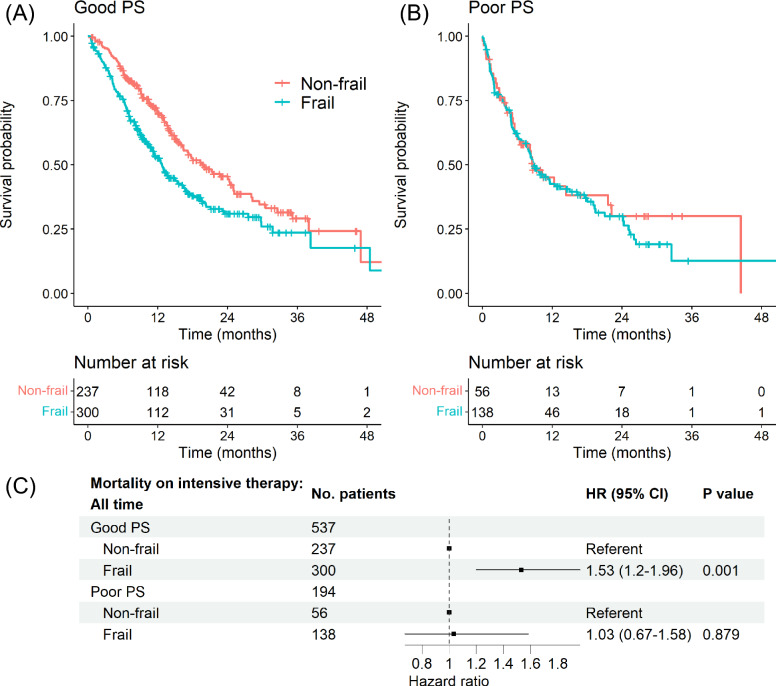
Stratified analysis by performance status: association of frailty with overall survival on intensive therapy. Overall survival in the intensive therapy cohort (*N* = 731) from the time of intensive therapy initiation. Intensive therapy is defined as first-line immune checkpoint inhibitor therapy with concurrent receipt of platinum-doublet chemotherapy and/or dual checkpoint blockade. Performance status (PS) is categorized as good (0–1) or poor (2 or greater) based on clinical notes at time of treatment initiation. Shown are Kaplan–Meier curves separated by frailty in patients with **A** good PS or **B** poor PS. **C** Forest plot of hazard ratio (HR) of overall survival estimated using multivariable Cox regression adjusting for age, gender, race/ethnicity, smoking status, cancer histology, stage at initial diagnosis, and PD-L1 score. Square symbols indicate the estimates of HR. Error bars indicate the 95% confidence interval (CI)

Unlike treatment selection, the impact of frailty on OS on intensive treatment differed depending on PS (Fig. 4). Frailty significantly predicted OS in patients with good PS (HR 1.53, 95% CI 1.20–1.96, *p* = 0.001). However, frailty did not significantly predict survival in patients with poor PS (HR 1.03, 95% CI 0.67–1.58, *p* = 0.879). The difference between the PS subgroups was most pronounced in the first 6 months after treatment initiation. During the first 6 months, frailty had a strong association with lower OS in the good PS subgroup (Good PS: HR 2.0, 95% CI 1.30–3.09, *p* = 0.002; Poor PS: HR 1.12, 95% CI 0.64–1.95, *p* = 0.693, Supplemental Fig. 4). Among patients who survived 6 months or more, frailty had reduced effect on survival (Good PS: HR 1.36, 95% CI 1–1.85, *p* = 0.047; Poor PS: HR 1.13, 95% CI 0.53–2.38, *p* = 0.751). Results for individual PD-L1 levels and ICI monotherapy were consistent with the primary analysis (Supplemental Figs. 5 and 6).

In our sensitivity analysis comparing OS between intensive treatment and ICI monotherapy, we found no survival difference in frail patients (Supplemental Fig. 7). Surprisingly, non-frail patients showed worse OS with intensive therapy, a trend that persisted across negative or low PD-L1 expressions (Supplemental Fig. 8). Therefore, this result should be cautiously interpreted due to potential confounding by indication, as patients with higher cancer burden and worse cancer-related prognosis may be chosen for more intensive treatment. This limitation is reduced in the primary analysis which only compares patients undergoing the same treatment.

In another sensitivity analysis, we tested whether age had a similar effect on survival as frailty. Patients aged 65 years or older tended to have reduced survival on intensive treatment (HR 1.22, 95% CI 0.94–1.57, *p* = 0.133). In contrast to frailty, the effect of age did not vary by PS in the first 6 months after treatment initiation (Supplemental Figs. 9 and 10). However, for those who survived 6 months after treatment, older patients with poor PS had worse survival than younger patients (Supplemental Figs. 9 and 10).

## Discussion

This population-based study provides contemporary real-world data on how frailty and PS affect immunotherapy use and outcomes in advanced NSCLC. We find that oncologists prefer ICI monotherapy for frail patients with both good and poor PS. Frailty was significantly associated with lower survival in patients with good PS who undergo intensive treatment, supporting concerns about increased toxicity in frail patients with minimal cancer symptoms. Surprisingly, among patients with poor PS, frailty was not associated with worse survival. This finding remained robust after adjustment for covariates such as PD-L1 and replicated across other immunotherapy regimens. This suggests that among this group, PS drives survival, rather than frailty. Prospective studies are needed before changing practice.

Due to perceived tolerability, ICI monotherapy is increasingly favored for frail patients [35] and those with poor PS [25, 26], often with minimal data [36]. Oncologists preferred ICI monotherapy for frail patients, irrespective of low or negative PD-L1 expression, indicating that concerns about frailty-related toxicity may have guided them away from the more intensive treatment that would be indicated based solely on PD-L1 levels. Frailty had a more significant impact on oncologists’ choice of administering ICI monotherapy with negative PD-L1 when PS was poor, and a lesser impact when PS was good. This indicates that oncologists might be concerned about additive treatment toxicity in patients with both frailty and poor PS. Additionally, they might opt for ICI monotherapy in frail patients with poor PS who would otherwise not receive any treatment, despite low or negative PD-L1 expression.

Our results provide context for recent conflicting phase III NSCLC trials including patients with poor PS. The IPSOS trial demonstrated a survival benefit of ICI monotherapy compared to single-agent chemotherapy in platinum-ineligible patients with poor PS [37]. In contrast, the energy trial did not show benefit of dual checkpoint blockade over platinum-doublet chemotherapy in patients with PS 2 [38]. Median OS varied between trials in both intervention (10.3 vs. 2.9 months) and control arms (9.2 vs. 6.1 months) [37, 38]. These discrepancies could stem from variations in patient selection, considering the heterogeneity of patients with poor PS. Importantly, frailty, which may contribute to poor PS, was not consistently measured. Our results suggest that in a real-world setting, frailty is unlikely to be the primary driver of low survival for patients with poor PS receiving intensive ICI therapy.

We found distinct effects of age and frailty on treatment selection and survival, indicating that the observed effects of frailty are not directly due to age. Unlike frailty, older age reduced survival for those with poor PS, especially those who survived beyond 6 months. Further studies are warranted to validate these findings and explore potential differing mechanisms of treatment resistance, such as immunosenescence in older adults [39].

Our study has several strengths. We replicate previously observed individual associations between frailty, poor PS, reduced survival and preference for less intense treatment obtained with in-person frailty assessments [4, 17] and manual PS abstraction [13, 18, 25, 26]. This supports the validity of computationally scalable measures of PS and frailty. To our knowledge, ours is the first population-based study to describe the combined impact of frailty and PS on immunotherapy selection for advanced NSCLC and demonstrate frailty has a diminished survival impact with immunotherapy for poor PS. Our results were consistent across multiple sensitivity analyses, supporting the robustness of our findings.

Despite these strengths, some limitations should be acknowledged. Despite adjustment for baseline covariates, there may be residual unmeasured confounding. Our binary classification of frailty may oversimplify the range seen in clinical practice, and frail patients selected for intensive treatment may differ from those not selected. However, frail patients on ICI monotherapy exhibit similar survival patterns, mitigating this limitation. We study only the impact of frailty on mortality; we do not address whether intensive treatment is superior to ICI monotherapy. Finally, quality-of-life outcomes, which can drive treatment decisions [17], were not measured in our study.

In conclusion, our real-world study demonstrates that frail patients with advanced NSCLC and poor PS often receive less intensive treatment, even when their PD-L1 status predicts suboptimal response to ICI monotherapy. However, we find no additive survival disadvantage on intensive treatment for patients who have both poor PS and are frail. Despite the lack of survival disadvantage, treating patients with poor PS may have other consequences. Prospective studies are needed to explore frailty’s impact on quality of life and to determine if ICI monotherapy can achieve comparable survival outcomes as intensive ICI treatment in cases of poor PS. Clinicians should consider both PS and frailty to balance the expected benefits of treatment with patient goals and preferences.

## Supplementary Information

Below is the link to the electronic supplementary material.

Supplementary file1 (DOCX 3017 KB)
