# Correction: The performance status gap in immunotherapy for frail patients with advanced non-small cell lung cancer

**DOI:** 10.1007/s00262-024-03875-3

**Published:** 2024-12-30

**Authors:** Julie Tsu-Yu Wu, June Corrigan, Chloe Su, Clark Dumontier, Jennifer La, Aparajita Khan, Shipra Arya, Alex H. S. Harris, Leah Backhus, Millie Das, Nhan V. Do, Mary T. Brophy, Summer S. Han, Michael Kelley, Nathanael R. Fillmore

**Affiliations:** 1 VA Palo Alto Healthcare System, Stanford University, Palo Alto, CA USA; 2 VA Boston Healthcare System, Boston, USA; 3 Stanford University, Palo Alto, CA USA; 4 VA Boston Healthcare System, Harvard Medical School, Boston, USA; 5 VA Boston Healthcare System, Boston University School of Medicine, Boston, USA; 6 Durham VA Healthcare System, Duke University, Durham, NC USA; 7 VA Boston Healthcare System, Dana-Farber Cancer Institute, Harvard Medical School, Boston, USA; 8 Massachusetts Veterans Epidemiology Research and Information Center, 150 S Huntington Ave, Boston, MA 02141 USA; 9 Present Address: Indian Institute of Technology Roorkee, Roorkee, India

**Correction to: Cancer Immunology, Immunotherapy (2024) 73:172.** 10.1007/s00262-024-03763-w

In the original version of this article, one author’s name was misspelled. Aparajita Khan was incorrectly written as Aparjita Khan.

The current affiliation 'Indian Institute of Technology Roorkee, Roorkee, India’ for author Aparajita Khan was missing.

