# Supplementary material for: The performance status gap in immunotherapy for frail patients with advanced non-small cell lung cancer
Source: Cancer Immunol Immunother. 2024 Jul 2;73(9):172. doi: 10.1007/s00262-024-03763-w (PMC11219626; doi:10.1007/s00262-024-03763-w)
Supplement: Supplementary file 1 — Supplementary file1 (DOCX 3017 KB) [file 262_2024_3763_MOESM1_ESM.docx]

**CONTENTS**

**Supplementary Methods**

Data sources and linkage

Adjustment factor variable definitions

NLP algorithms for stage and performance status

Health deficit domains included in VA frailty index calculation

Missing data handling

**Supplementary Tables**

Supplemental table 1. Missingness table

Supplemental table 2. Patient characteristics by frailty

Supplemental table 3. Patient characteristics by performance status

Supplemental table 4. Treatment regimens frequency table

Supplemental table 5. Multivariable logistic regression for evaluating treatment selection

Supplemental table 6. Multivariable logistic regression for evaluating treatment selection in the imputed dataset

Supplemental table 7. Multivariable Cox regression for evaluating overall survival on intensive treatment

Supplemental table 8. Multivariable Cox regression for evaluating overall survival on intensive treatment in imputed dataset

**Supplementary Figures**

Supplemental figure 1. Association of frailty with intensive treatment selection when stratified by performance status and PD-L1

Supplemental figure 2. Association of age with intensive treatment selection when stratified by performance status

Supplemental figure 3. Kaplan-Meier curves of all frailty and performance status subgroups in the intensive therapy cohort

Supplemental figure 4. Association of frailty with overall survival when stratified by performance status in the 0-6 month time period and 6+ months

Supplemental figure 5. Kaplan-Meier curves and association of frailty with overall survival on intensive therapy across PD-L1 expression levels

Supplemental figure 6. Stratified analysis by performance status: association of frailty with overall survival on ICI monotherapy.

Supplemental figure 7. Kaplan-Meier curves for overall survival on intensive therapy among patients aged <65y vs. 65+

Supplemental figure 8. Association of age with overall survival on intensive therapy when stratified by performance status

**Supplementary Methods**

**Data Sources and Linkage**

This study utilizes patient-level data from the Veterans Affairs (VA) healthcare system, the largest integrated healthcare system in the United States, consisting of 171 medical centers and 1,113 outpatient clinics across the nation.

To link the data at the patient level, we matched unique identifiers between electronic health records in the VA Corporate Data Warehouse (CDW), the national VA Cancer Registry, and the VA National Precision Oncology Program^1^.

Data extracted from the VA Cancer Registry included cancer histology, American Joint Committee on Cancer (AJCC) staging and demographics. Data extracted from the VA Precision Oncology Program included cancer histology and PD-L1 score. From the CDW, we extracted data on demographics, mortality, clinical encounters, medical history, problem lists, medications, International Classification of Diseases Clinical Modification (ICD-9-CM and ICD-10-CM) diagnosis and procedure codes, and clinic notes. Missing structured data for cancer stage and PD-L1 score was supplemented by natural language processing (NLP) from clinical notes as described below.

**Adjustment factor variable definitions**

Age:

Age in years was extracted directly from the CDW at the time of treatment initiation.

Sex:

Sex was extracted directly from the CDW at the time of treatment initiation.

Race/Ethnicity:

Race/ethnicity was extracted from the VA Cancer Registry and CDW and recoded as Non-Hispanic White, Non-Hispanic Black, Hispanic, Non-Hispanic Asian, Pacific Islander, Alaskan Natives, American Indian (altogether classified as API), or Other/Unknown. Groups with representation <10% were grouped together as “Other.”

Smoking status:

Smoking status (current, former, never) was extracted directly from the CDW at the time of treatment initiation.

Cancer site/histology subtype:

Cancer site and histology were recoded from VA Cancer Registry at the time of diagnosis according to the SEER standard ICD-O-3 codes as previously defined^2^.

Cancer Stage:

AJCC cancer staging (I, II, III, IV, unknown) at the time of cancer diagnosis was extracted from the VA Cancer Registry. If stage was unknown from the registry, NLP was used to derive stage from clinical notes. If multiple stages from notes were found, the most advanced stage was used.

PD-L1 score:

The numeric PD-L1 score was extracted directly from VA Precision Oncology Program data and classified as follows: 0 is negative, 1-49 is low, and 50 or greater is high. If the PD-L1 value was not available from VA Precision Oncology data, it was extracted with NLP as previously described^3^.

**NLP algorithms for stage and performance status**

For efficient NLP from patient clinical notes, we have derived a workflow for identifying oncology clinical notes as previously described^3^. From these notes we have developed a customized extraction algorithm for stage and performance status based on pattern matching.

**Health deficit domains included in VA frailty index calculation**

Health deficits included in the VA frailty index were defined as previously described^4^. Each health deficit was identified based on the presence of associated International Classification of Diseases (ICD-10), Current Procedural Terminology (CPT), or Healthcare Common Procedure Coding System (HCPCS) codes. Included health deficits are as follows: Atrial fibrillation, Anemia, Anxiety, Arthritis, Coronary artery disease, Cancer, Chronic pain, Cerebrovascular disease, Dementia, Depression, Diabetes, Durable medical equipment, Falls, Fatigue, Failure to thrive, Gait abnormality, Hearing impairment/loss, Heart failure, Hypertension, Incontinence, Kidney disease, Liver disease or cirrhosis, Lung disease (COPD, asthma), Muscular issue, Osteoporosis, Parkinson's disease, Peripheral neuropathy, Peripheral vascular disease, Thyroid disease, Vision comorbidity, Weight loss.

**Missing data handling**

Missing structured data for stage and PD-L1 was supplemented by NLP to extract this information from clinical notes. When both structured and NLP data were available, we used the value closest to the date of ICI initiation. After this combined approach, the missing rate of variables was <10%, except for the following: histology (22.0% missing) and stage (21.7% missing) (supplemental table 1). Frailty does not have missing values due to the nature of its definition by administrative codes. We conducted our primary analysis with unknown values as a separate category for each covariate. If the unknown category was <10% of the cohort, it was combined with another, specified category to form a combined category (e.g., “other/unknown”).

**REFERENCES**

**1**. Kelley MJ: VA National Precision Oncology Program. Fed Pract 37:S22–S27, 2020

**2**. Lewis DR, Check DP, Caporaso NE, et al: US lung cancer trends by histologic type. Cancer 120:2883–2892, 2014

**3**. Lin E, Zwolinski R, Wu J, et al: Machine Learning-Based Natural Language Processing to Extract PD-L1 Expression Levels from Clinical Notes

**4**. Cheng D, DuMontier C, Yildirim C, et al: Updating and Validating the U.S. Veterans Affairs Frailty Index: Transitioning From ICD-9 to ICD-10. J Gerontol A Biol Sci Med Sci 76:1318–1325, 2021

**Supplemental table 1. Missingness table**

| Variable | Missing rate, n (%) |
| --- | --- |
| Histology | 341 (22.0) |
| Stage | 335 (21.7) |
| Smoking status | 58 (3.8) |
| Race/ethnicity | 57 (3.8) |

**Supplemental table 2. Patient characteristics by frailty**

|  |  | |  | **Frailty** | | | | | |
| --- | --- | --- | --- | --- | --- | --- | --- | --- | --- |
|  | **Total** | |  | **Non-frail*** | | |  | **Frail*** | |
|  | N | (%) |  | N | (%) | |  | N | (%) |
| **Total No. of Patients** | 1547 |  |  | 523 | |  |  | 1024 |  |
| **Demographics** |  |  |  |  | |  |  |  |  |
| Age at treatment initiation (years) | | | |  | |  |  |  |  |
| Mean (SD) | 70.5 | 7.43 |  | 68.7 | | 7.55 |  | 71.46 | 7.20 |
| Age strata |  |  |  |  | |  |  |  |  |
| <65 years old | 339 | 21.9 |  | 156 | | 29.8 |  | 183 | 17.9 |
| 65+ years old | 1208 | 78.1 |  | 367 | | 70.2 |  | 841 | 82.1 |
| Sex |  |  |  |  | |  |  |  |  |
| Male | 1496 | 96.7 |  | 497 | | 95.0 |  | 999 | 97.6 |
| Female | 51 | 3.3 |  | 26 | | 5.0 |  | 25 | 2.4 |
| Race |  |  |  |  | |  |  |  |  |
| Non-Hispanic White | 1089 | 70.4 |  | 377 | | 72.1 |  | 712 | 69.5 |
| Black | 331 | 21.4 |  | 105 | | 20.1 |  | 226 | 22.1 |
| Other/unknown | 127 | 8.2 |  | 41 | | 7.8 |  | 86 | 8.4 |
| Smoking status† |  |  |  |  | |  |  |  |  |
| Current | 806 | 52.1 |  | 285 | | 54.5 |  | 521 | 50.9 |
| Former | 649 | 42.0 |  | 198 | | 37.9 |  | 451 | 44.0 |
| Never/unknown | 92 | 5.9 |  | 40 | | 7.6 |  | 52 | 5.1 |
| **Clinical characteristics**§ |  |  |  |  | |  |  |  |  |
| Performance status |  |  |  |  | |  |  |  |  |
| 0-1 | 1024 | 66.2 |  | 400 | | 76.5 |  | 624 | 60.9 |
| 2 or greater | 523 | 33.8 |  | 123 | | 23.5 |  | 400 | 39.1 |
| **Tumor characteristics** | | |  |  | |  |  |  |  |
| Histology |  |  |  |  | |  |  |  |  |
| Adenocarcinoma | 731 | 47.3 |  | 266 | | 50.9 |  | 465 | 39.1 |
| Squamous cell | 321 | 20.7 |  | 95 | | 18.2 |  | 226 | 22.1 |
| Other/unknown | 495 | 32.0 |  | 162 | | 31.0 |  | 333 | 32.5 |
| Stage at initial diagnosis |  |  |  |  | |  |  |  |  |
| I | 205 | 13.3 |  | 52 | | 9.9 |  | 153 | 14.9 |
| II | 90 | 5.8 |  | 24 | | 4.6 |  | 66 | 6.4 |
| III | 227 | 14.7 |  | 71 | | 13.6 |  | 156 | 15.2 |
| IV | 690 | 44.6 |  | 275 | | 52.6 |  | 415 | 40.5 |
| Unknown | 335 | 21.7 |  | 101 | | 19.3 |  | 234 | 22.9 |
| PD-L1 expression‡ |  |  |  |  | |  |  |  |  |
| Negative | 457 | 29.5 |  | 164 | | 31.4 |  | 293 | 28.6 |
| Low | 511 | 33.0 |  | 165 | | 31.5 |  | 346 | 33.8 |
| High | 579 | 37.4 |  | 194 | | 37.1 |  | 385 | 37.6 |
| Treatment** |  |  |  |  | |  |  |  |  |
| Non-intensive | 816 | 52.7 |  | 230 | | 44.0 |  | 586 | 57.2 |
| Intensive | 731 | 47.3 |  | 293 | | 56.0 |  | 438 | 42.8 |

Abbreviations: ICI, immune checkpoint inhibitor; SD, standard deviation

* Frail was defined using a prespecified cutoff of 0.2 as measured at time of ICI treatment initation, as detailed in Methods.

** Intensive ICI treatment was defined as receipt of first-line dual checkpoint blockade or chemoimmunotherapy, as detailed in Methods. Non-intensive therapy was defined as receipt of first-line ICI without dual checkpoint blockade or chemotherapy.

† Smoking status is at time of ICI treatment initiation

§ Clinical characteristics (performance status) were measured at the time of ICI treatment initiation

‡ PD-L1 expression levels were defined as follows: Negative 0%, Low 1-49%, High 50% or greater

**Supplemental table 3. Patient characteristics by performance status**

|  |  | |  | **Performance status**§ | | | | |
| --- | --- | --- | --- | --- | --- | --- | --- | --- |
|  | **Total** | |  | **0-1** | |  | **2+** | |
|  | N | (%) |  | N | (%) |  | N | (%) |
| **Total No. of Patients** | 1547 |  |  | 1024 |  |  | 523 |  |
| **Demographics** |  |  |  |  |  |  |  |  |
| Age at treatment initiation (years) | | | |  |  |  |  |  |
| Mean (SD) | 70.5 | 7.43 |  | 69.9 | 7.36 |  | 71.8 | 7.41 |
| Age strata |  |  |  |  |  |  |  |  |
| <65 years old | 339 | 21.9 |  | 249 | 24.3 |  | 90 | 17.2 |
| 65+ years old | 1208 | 78.1 |  | 775 | 75.7 |  | 433 | 82.8 |
| Sex |  |  |  |  |  |  |  |  |
| Male | 1496 | 96.7 |  | 987 | 96.4 |  | 509 | 97.3 |
| Female | 51 | 3.3 |  | 37 | 3.6 |  | 14 | 2.7 |
| Race |  |  |  |  |  |  |  |  |
| Non-Hispanic White | 1089 | 70.4 |  | 730 | 71.3 |  | 359 | 68.6 |
| Black | 331 | 21.4 |  | 210 | 20.5 |  | 121 | 23.1 |
| Other/unknown | 127 | 8.2 |  | 84 | 8.2 |  | 43 | 8.2 |
| Smoking status† |  |  |  |  |  |  |  |  |
| Current | 806 | 52.1 |  | 538 | 52.5 |  | 268 | 51.2 |
| Former | 649 | 42.0 |  | 422 | 41.2 |  | 227 | 43.4 |
| Never/unknown | 92 | 5.9 |  | 64 | 6.2 |  | 28 | 5.4 |
| **Clinical characteristics**§ |  |  |  |  |  |  |  |  |
| Frailty |  |  |  |  |  |  |  |  |
| Non-frail | 523 | 33.8 |  | 400 | 39.1 |  | 123 | 23.5 |
| Frail | 1024 | 66.2 |  | 624 | 60.9 |  | 400 | 76.5 |
| **Tumor characteristics** |  |  |  |  |  |  |  |  |
| Histology |  |  |  |  |  |  |  |  |
| Adenocarcinoma | 731 | 47.3 |  | 491 | 47.9 |  | 240 | 45.9 |
| Squamous cell | 321 | 20.7 |  | 193 | 18.8 |  | 128 | 24.5 |
| Other/unknown | 495 | 32.0 |  | 340 | 33.2 |  | 155 | 29.6 |
| Stage at initial diagnosis |  |  |  |  |  |  |  |  |
| I | 205 | 13.3 |  | 140 | 13.7 |  | 65 | 12.4 |
| II | 90 | 5.8 |  | 63 | 6.2 |  | 27 | 5.2 |
| III | 227 | 14.7 |  | 151 | 14.7 |  | 76 | 14.5 |
| IV | 690 | 44.6 |  | 437 | 42.7 |  | 253 | 48.4 |
| Unknown | 335 | 21.7 |  | 233 | 22.8 |  | 102 | 19.5 |
| PD-L1 expression‡ |  |  |  |  |  |  |  |  |
| Negative | 457 | 29.5 |  | 330 | 32.2 |  | 127 | 24.3 |
| Low | 511 | 33.0 |  | 350 | 34.2 |  | 161 | 30.8 |
| High | 579 | 37.4 |  | 344 | 33.6 |  | 235 | 44.9 |
| Treatment** |  |  |  |  |  |  |  |  |
| Non-intensive | 816 | 52.7 |  | 487 | 47.6 |  | 329 | 62.9 |
| Intensive | 731 | 47.3 |  | 537 | 52.4 |  | 194 | 37.1 |

Abbreviations: ICI, immune checkpoint inhibitor; SD, standard deviation

* Frail was defined using a prespecified cutoff of 0.2 as measured at time of ICI treatment initation, as detailed in Methods.

** Intensive ICI treatment was defined as receipt of first-line dual checkpoint blockade or chemoimmunotherapy, as detailed in Methods. Non-intensive therapy was defined as receipt of first-line ICI without dual checkpoint blockade or chemotherapy.

† Smoking status is at time of ICI treatment initiation

§ Performance status was measured at the time of ICI treatment initiation, as detailed in Methods.

‡ PD-L1 expression levels were defined as follows: Negative 0%, Low 1-49%, High 50% or greater

**Supplemental table 4. Treatment regimens frequency table**

| **Treatment regimen** | **N (%)** |
| --- | --- |
| **Intensive therapy** |  |
| Carboplatin+pemetrexed+pembrolizumab | 452 (61.8) |
| Carboplatin+paclitaxel+pembrolizumab | 206 (28.2) |
| Carboplatin+etoposide+atezolizumab | 22 (3.0) |
| Other carboplatin-based doublet+ICI | 16 (2.2) |
| Cisplatin-based doublet+ICI | 7 (1.0) |
| Ipilimumab+nivolumab | 14 (1.9) |
| Platinum doublet+ipilimumab+nivolumab | 14 (1.9) |
| **Non-intensive therapy** |  |
| Pembrolizumab | 679 (83.2) |
| Nivolumab | 106 (13.0) |
| Atezolizumab | 31 (3.8) |

Abbreviations: ICI, immune checkpoint inhibitor

**Supplemental table 5. Multivariable logistic regression for evaluating treatment selection**

| Characteristics | Odds ratio (95% CI) | p-value |
| --- | --- | --- |
| Frailty§ |  |  |
| Non-frail | Reference |  |
| Frail | 0.67 | 9.52E-04 |
| Performance status§ |  |  |
| 0-1 | Reference |  |
| 2 or greater | 0.60 | 2.95E-05 |
| Age at treatment initiation |  |  |
| <65 years old | Reference |  |
| 65+ years old | 0.87 | 0.341 |
| Sex |  |  |
| Male | Reference |  |
| Female | 0.66 | 0.186 |
| Race |  |  |
| Non-Hispanic white | Reference |  |
| Black | 0.76 | 0.050 |
| Other/unknown | 1.24 | 0.307 |
| Smoking status† |  |  |
| Current | Reference |  |
| Former | 0.90 | 0.365 |
| Never/unknown | 1.56 | 0.071 |
| Histology |  |  |
| Adenocarcinoma | Reference |  |
| Squamous cell | 0.76 | 0.073 |
| Other/unknown | 0.91 | 0.551 |
| Stage at initial diagnosis |  |  |
| IV | Reference |  |
| I | 0.56 | 7.85E-04 |
| II | 0.19 | 1.82E-09 |
| III | 0.29 | 4.38E-12 |
| Unknown | 0.73 | 0.079 |
| PD-L1 expression‡ |  |  |
| Negative | Reference |  |
| Low | 0.73 | 0.022 |
| High | 0.237 | 7.28E-24 |

† Smoking status is at time of ICI treatment initiation

§ Frailty and performance status was measured at the time of ICI treatment initiation, as detailed in Methods.

‡ PD-L1 expression levels were defined as follows: Negative 0%, Low 1-49%, High 50% or greater

**Supplemental table 6. Multivariable logistic regression for evaluating treatment selection in the imputed dataset**

| Characteristics | Odds ratio (95% CI) | p-value |
| --- | --- | --- |
| Frailty§ |  |  |
| Non-frail | Reference |  |
| Frail | 0.70 | 0.004 |
| Performance status§ |  |  |
| 0-1 | Reference |  |
| 2 or greater | 0.59 | 2.53E-05 |
| Age at treatment initiation |  |  |
| <65 years old | Reference |  |
| 65+ years old | 0.86 | 0.275 |
| Sex |  |  |
| Male | Reference |  |
| Female | 0.64 | 0.163 |
| Race |  |  |
| Non-Hispanic white | Reference |  |
| Black | 0.73 | 0.029 |
| Hispanic or Latino | 1.80 | 0.096 |
| Other | 1.32 | 0.564 |
| Smoking status† |  |  |
| Current | Reference |  |
| Former | 0.90 | 0.394 |
| Never | 1.24 | 0.580 |
| Histology |  |  |
| Adenocarcinoma | Reference |  |
| Squamous cell | 0.75 | 0.128 |
| Other | 1.01 | 0.964 |
| Stage at initial diagnosis |  |  |
| IV | Reference |  |
| I | 0.49 | 2.71E-05 |
| II | 0.16 | 5.79E-06 |
| III | 0.28 | 1.39E-10 |
| PD-L1 expression‡ |  |  |
| Negative | Reference |  |
| Low | 0.69 | 0.010 |
| High | 0.22 | 0 |

† Smoking status is at time of ICI treatment initiation

§ Frailty and performance status was measured at the time of ICI treatment initiation, as detailed in Methods.

‡ PD-L1 expression levels were defined as follows: Negative 0%, Low 1-49%, High 50% or greater

**Supplemental table 7. Multivariable Cox regression for evaluating overall survival on intensive treatment**

| Characteristics | Hazard ratio (95% CI) | p-value |
| --- | --- | --- |
| Frailty§ |  |  |
| Non-frail | Reference |  |
| Frail | 1.36 | 0.005 |
| Performance status§ |  |  |
| 0-1 | Reference |  |
| 2 or greater | 1.52 | 1.84E-04 |
| Age at treatment initiation |  |  |
| <65 years old | Reference |  |
| 65+ years old | 1.22 | 0.133 |
| Sex |  |  |
| Male | Reference |  |
| Female | 0.80 | 0.523 |
| Race |  |  |
| Non-Hispanic white | Reference |  |
| Black | 0.87 | 0.300 |
| Other/unknown | 0.58 | 0.005 |
| Smoking status† |  |  |
| Current | Reference |  |
| Former | 1.02 | 0.820 |
| Never/unknown | 1.04 | 0.851 |
| Histology |  |  |
| Adenocarcinoma | Reference |  |
| Squamous cell | 1.13 | 0.362 |
| Other/unknown | 0.88 | 0.432 |
| Stage at initial diagnosis |  |  |
| IV | Reference |  |
| I | 0.75 | 0.079 |
| II | 0.79 | 0.490 |
| III | 0.78 | 0.187 |
| Unknown | 1.30 | 0.121 |
| PD-L1 expression‡ |  |  |
| Negative | Reference |  |
| Low | 0.79 | 0.036 |
| High | 0.68 | 0.004 |

† Smoking status is at time of ICI treatment initiation

§ Frailty and performance status was measured at the time of ICI treatment initiation, as detailed in Methods.

‡ PD-L1 expression levels were defined as follows: Negative 0%, Low 1-49%, High 50% or greater

**Supplemental table 8. Multivariable Cox regression for evaluating overall survival on intensive treatment in imputed dataset**

| Characteristics | Hazard ratio (95% CI) | p-value |
| --- | --- | --- |
| Frailty§ |  |  |
| Non-frail | Reference |  |
| Frail | 1.44 | 2.94E-06 |
| Performance status§ |  |  |
| 0-1 | Reference |  |
| 2 or greater | 1.84 | 0 |
| Age at treatment initiation |  |  |
| <65 years old | Reference |  |
| 65+ years old | 1.18 | 0.058 |
| Sex |  |  |
| Male | Reference |  |
| Female | 0.75 | 0.19 |
| Race |  |  |
| Non-Hispanic white | Reference |  |
| Black | 0.77 | 0.003 |
| Hispanic or Latino | 0.62 | 0.021 |
| Other | 0.58 | 0.082 |
| Smoking status† |  |  |
| Current | Reference |  |
| Former | 0.91 | 0.186 |
| Never | 0.79 | 0.361 |
| Histology |  |  |
| Adenocarcinoma | Reference |  |
| Squamous cell | 1.10 | 0.295 |
| Other | 0.97 | 0.830 |
| Stage at initial diagnosis |  |  |
| IV | Reference |  |
| I | 0.77 | 0.013 |
| II | 0.78 | 0.126 |
| III | 0.87 | 0.177 |
| PD-L1 expression‡ |  |  |
| Negative | Reference |  |
| Low | 0.88 | 0.15 |
| High | 0.72 | 1.50E-04 |

† Smoking status is at time of ICI treatment initiation

§ Frailty and performance status was measured at the time of ICI treatment initiation, as detailed in Methods.

‡ PD-L1 expression levels were defined as follows: Negative 0%, Low 1-49%, High 50% or greater

**Supplemental figure 1. Association of frailty with intensive treatment selection when stratified by performance status and PD-L1.** First-line immune checkpoint inhibitor (ICI) treatment selection in the all-treatment cohort (N=1547) and stratified by both performance status (PS) and PD-L1 expression level: (A) negative (0%), (B) low (1-49%), and (C) high (≥50%). Forest plot of odds ratio (OR) for treatment selection estimated using multivariable logistic regression adjusting for age, gender, race/ethnicity, smoking status, cancer histology, and stage at initial diagnosis, stratified by PS and PD-L1 expression: (D) negative (0%), (E) low (1-49%), and (F) high (≥50%). **Square** symbols indicate the estimates of OR. **Error bars** indicate the 95% confidence interval (CI).


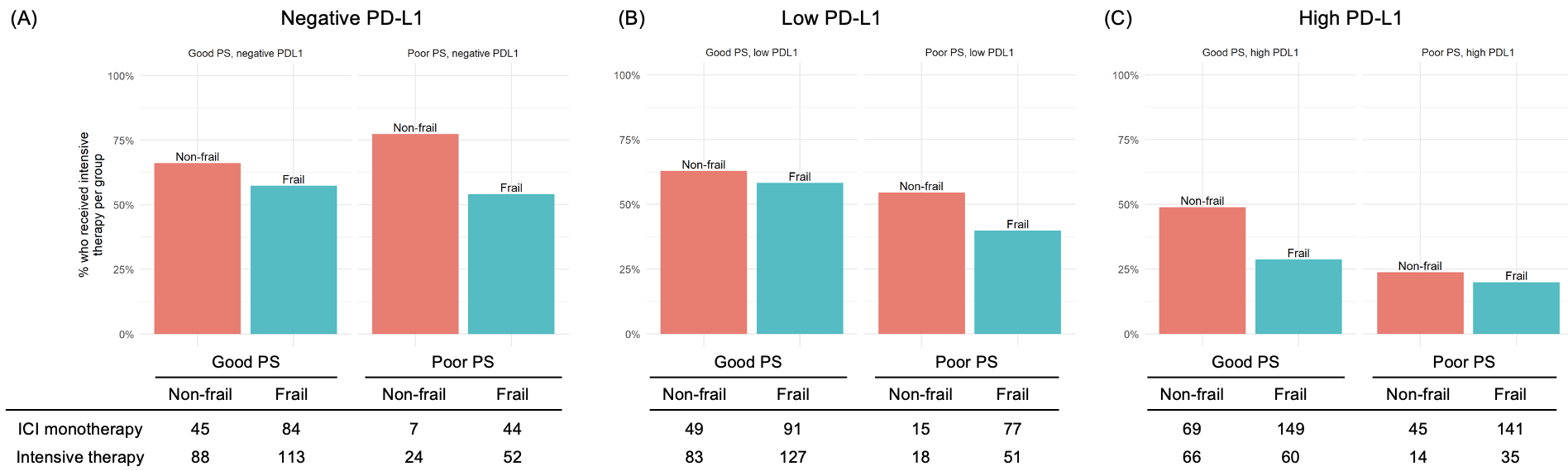


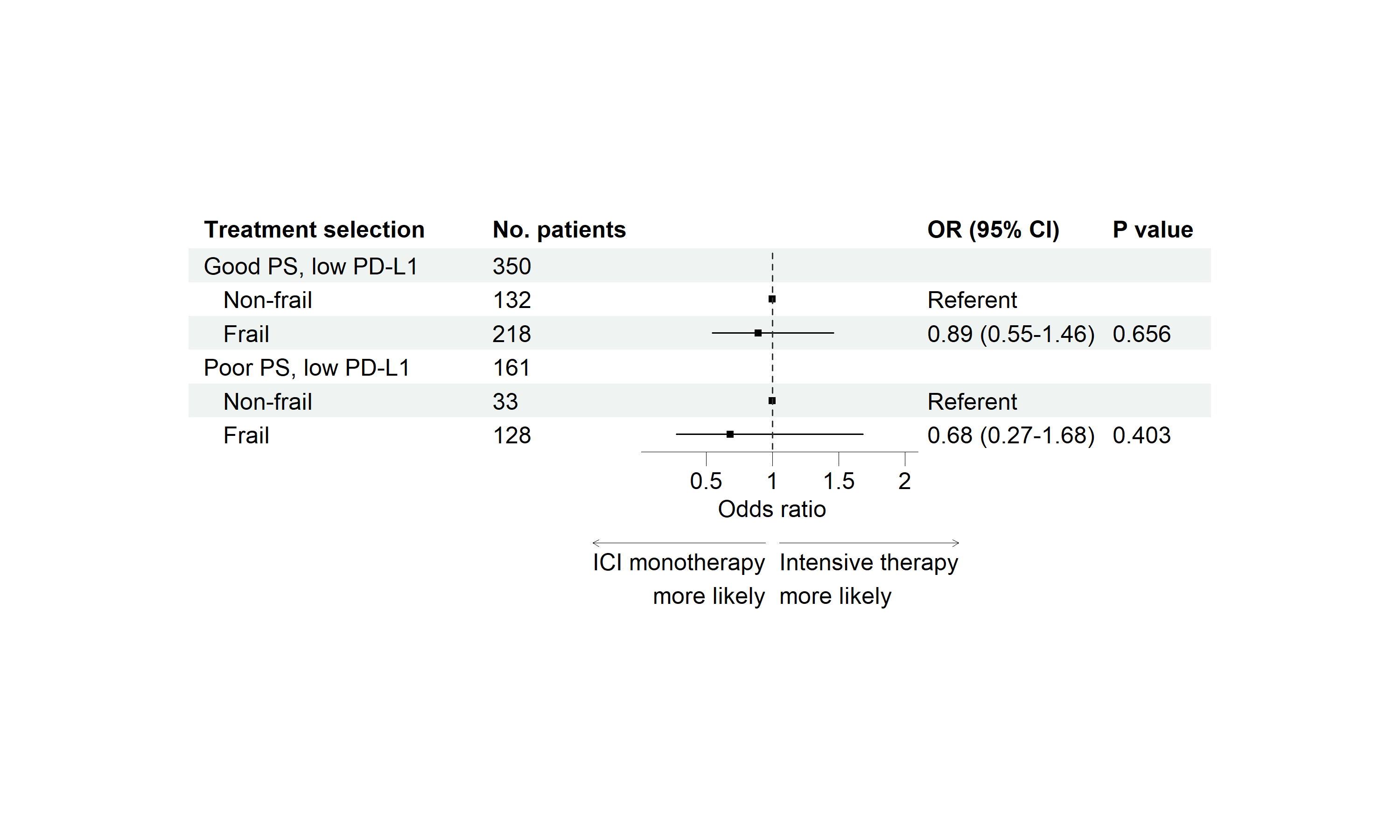


(E)

(D)

(F)


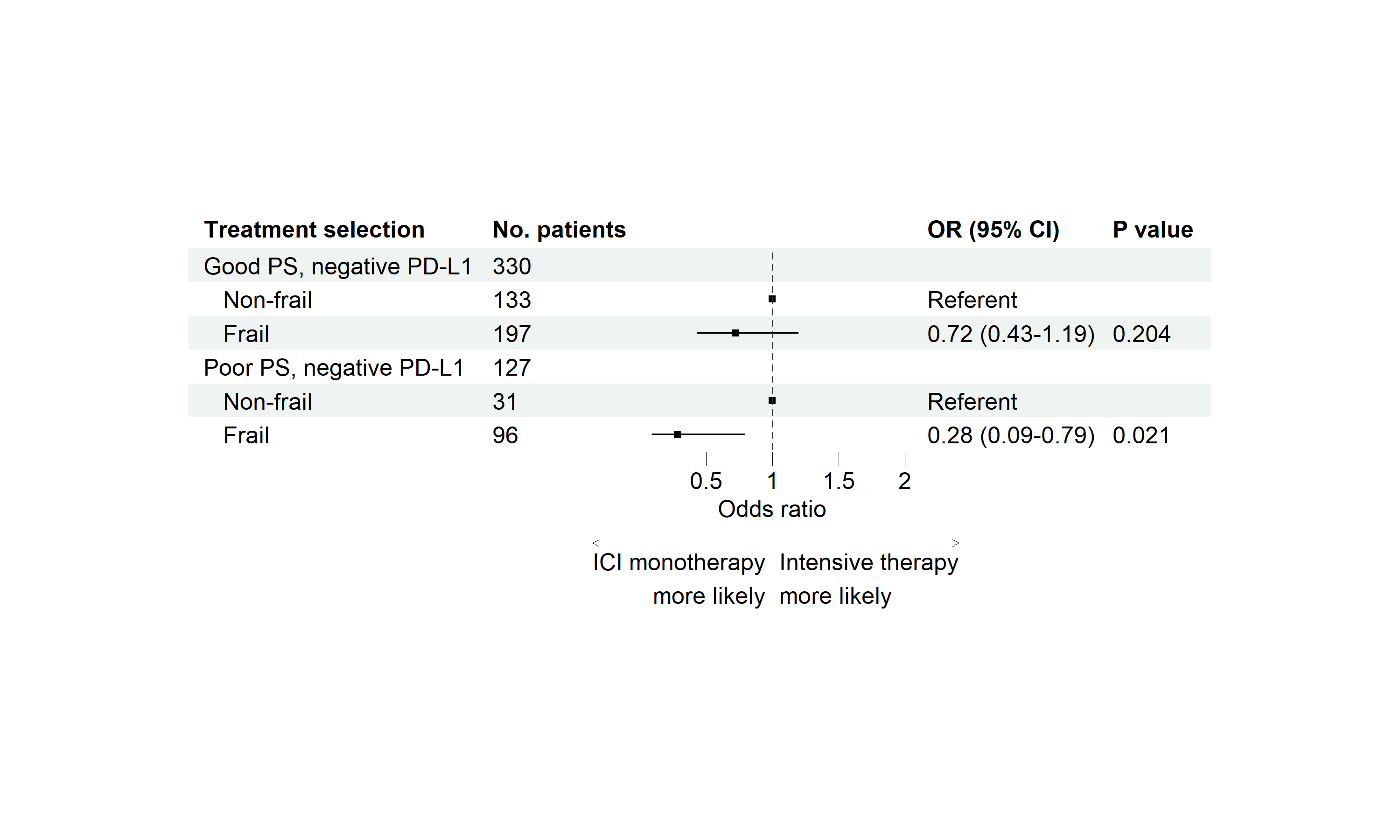

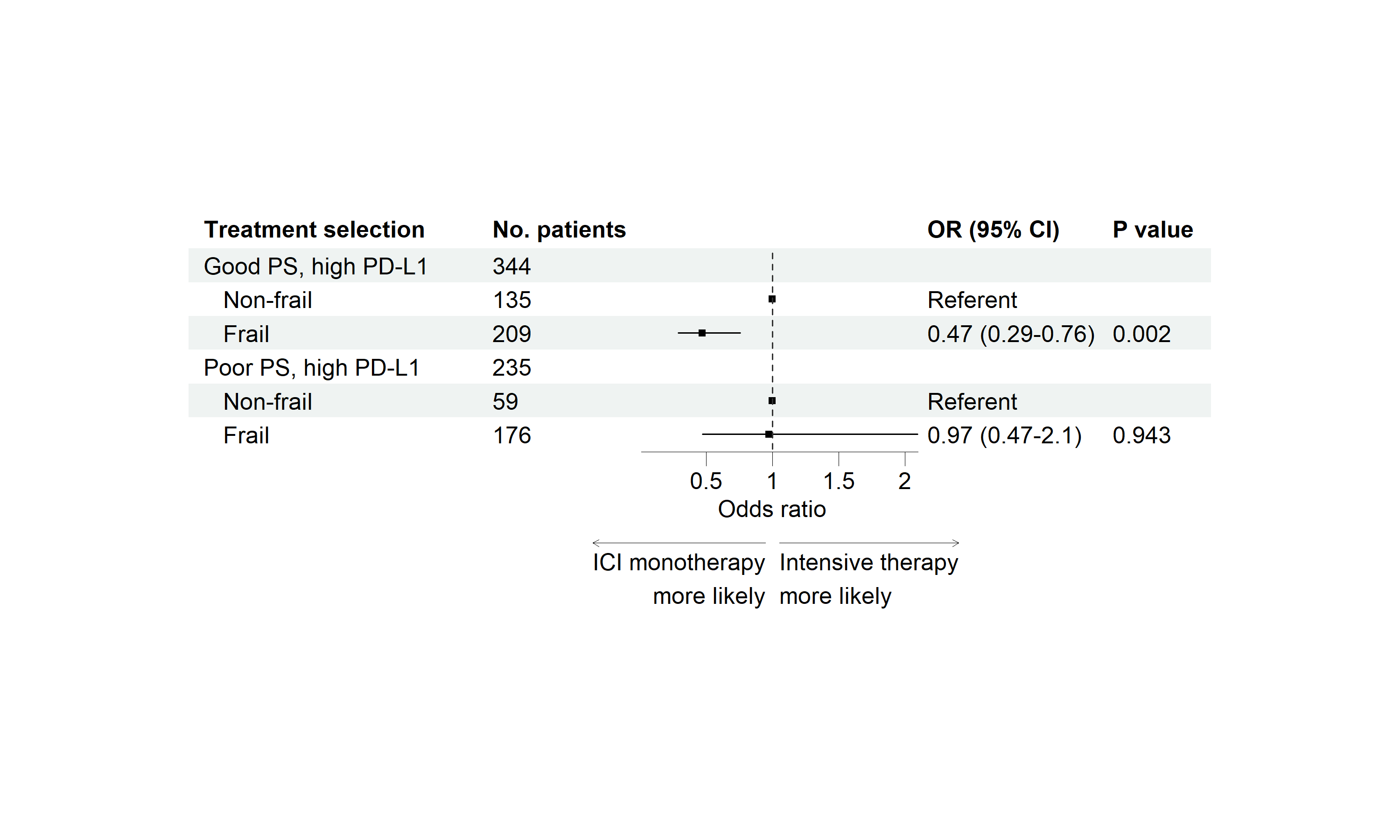


**Supplemental figure 2. Association of age with intensive treatment selection when stratified by performance status.** Forest plot of odds ratio (OR) for treatment selection in the all-treatment cohort (N=1547) estimated using multivariable logistic regression adjusting for fraily, gender, race/ethnicity, smoking status, cancer histology, stage at initial diagnosis, and PD-L1 score. Performance status (PS) is categorized as good (0-1) or poor (2 or greater) based on clinical notes at time of treatment initiation. Intense therapy is defined as first-line immune checkpoint inhibitor (ICI) with concurrent receipt of platinum-doublet chemotherapy and/or dual checkpoint blockade. Non-intense therapy was defined as receipt of first-line ICI without dual checkpoint blockade or chemotherapy. **Square** symbols indicate the estimates of OR. **Error bars** indicate the 95% confidence interval (CI).


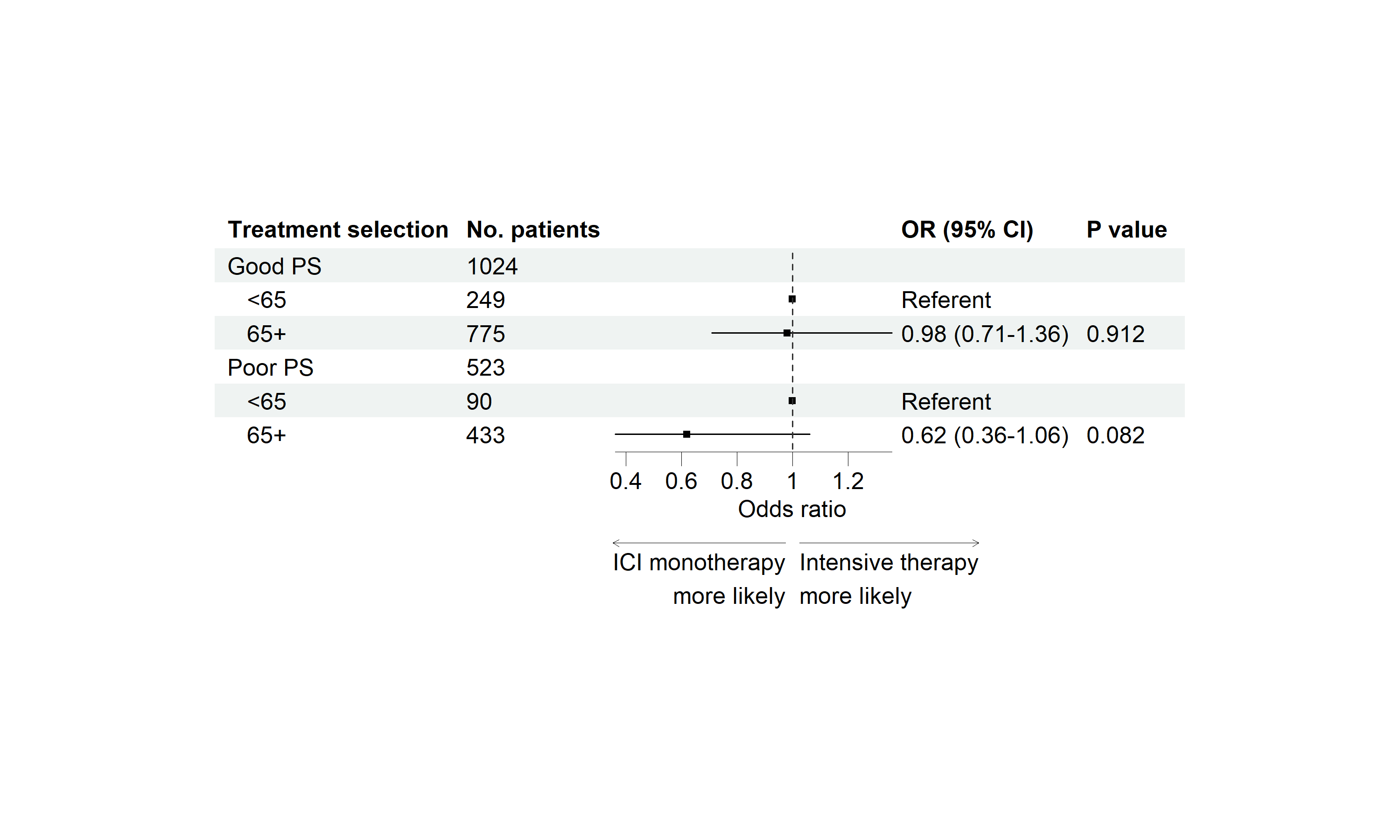


**Supplemental figure 3. Kaplan-Meier curves of all frailty and performance status subgroups in the intensive therapy cohort.** Overall survival in the intensive therapy cohort (N=731) from the time of intensive therapy initiation. Intensive therapy is defined as first-line immune checkpoint inhibitor therapy with concurrent receipt of platinum-doublet chemotherapy and/or dual checkpoint blockade. (A) Kaplan-Meier curves separated by frailty at treatment initiation, (B) separated by performance status (PS), and (C) separated by frailty and PS. PS is categorized as good (0-1) or poor (2 or greater) based on clinical notes at time of treatment initiation.


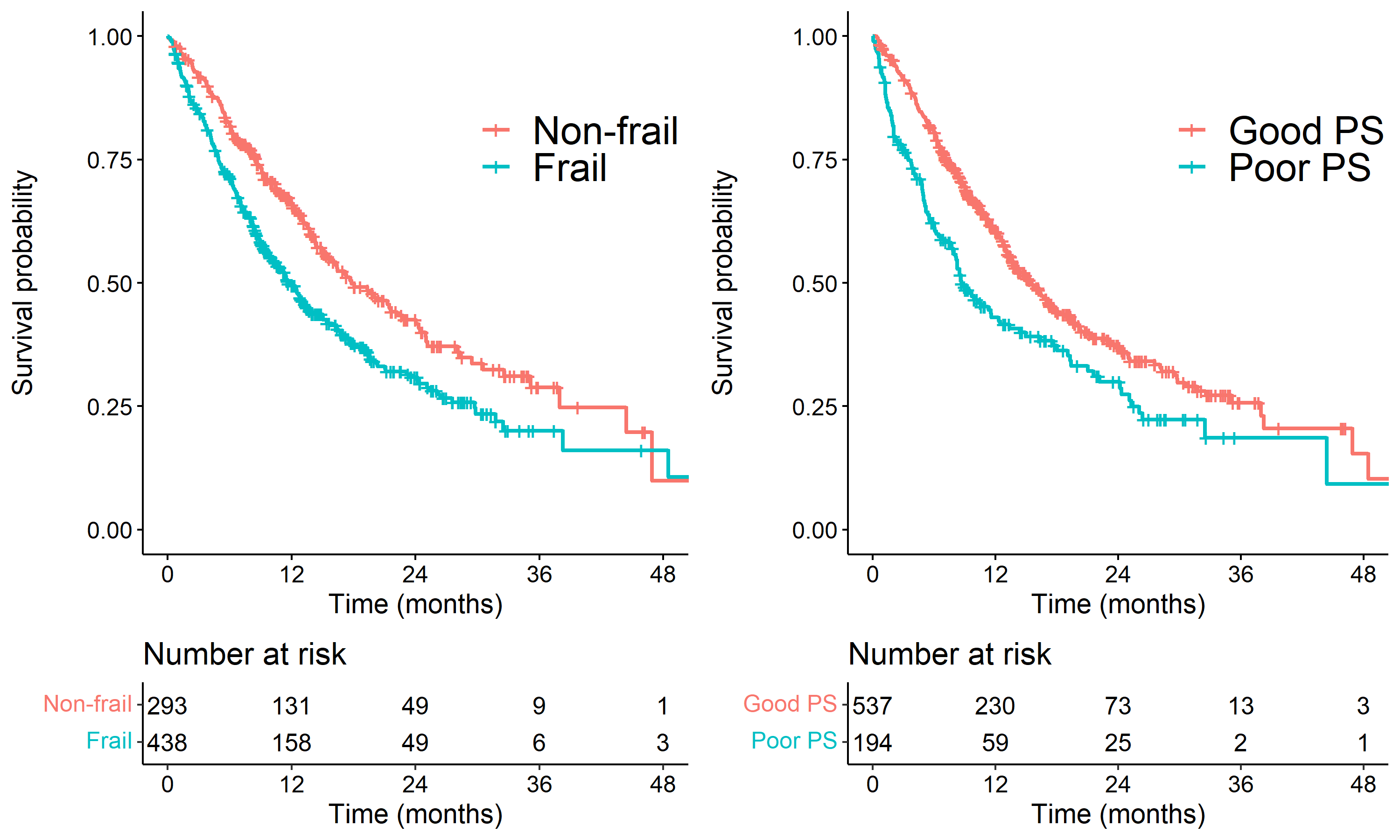


(A)

(B)


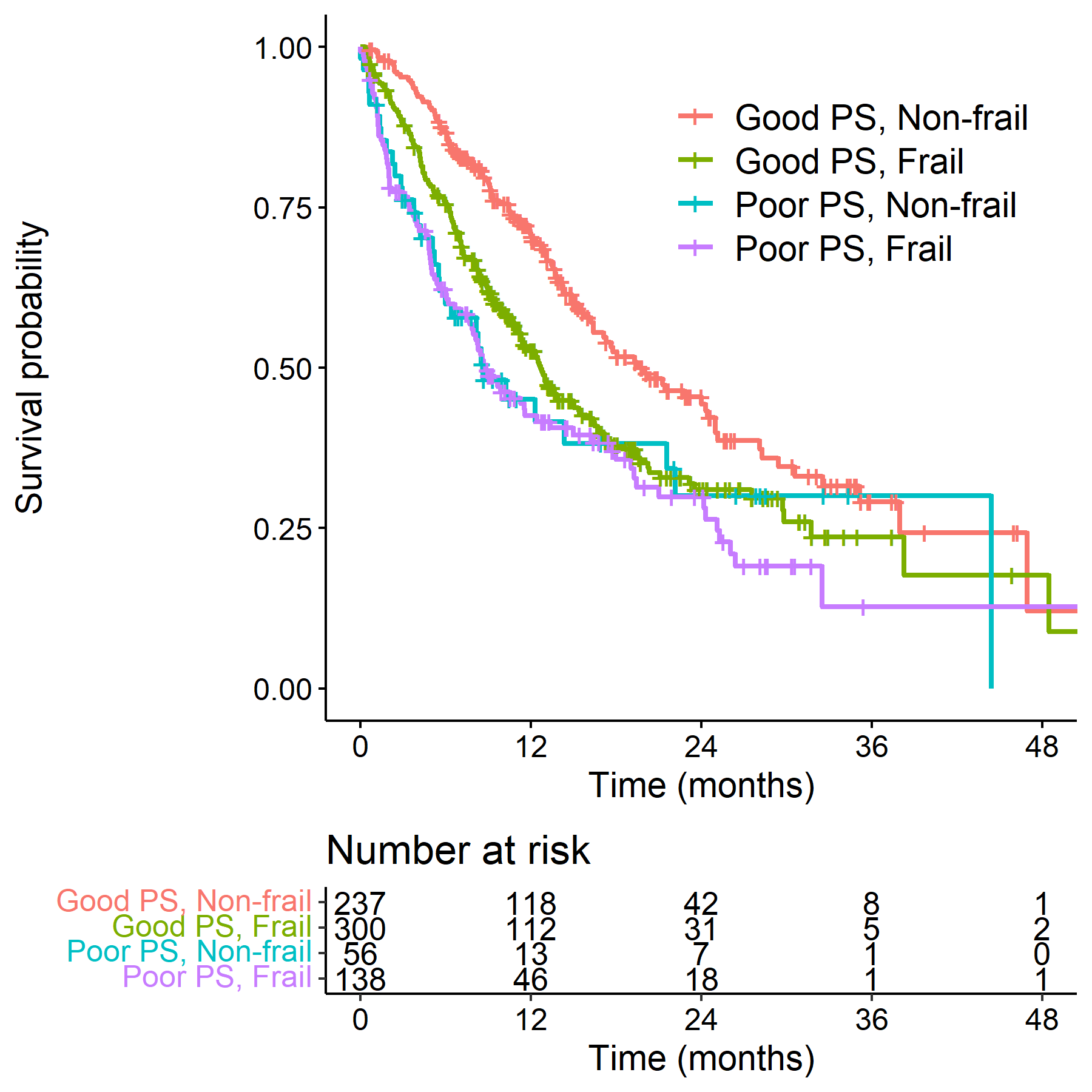


(C)

**Supplemental figure 4. Association of frailty with overall survival when stratified by performance status in the 0-6 month time period and 6+ months.** Forest plot of hazard ratio (HR) of overall survival in the intensive therapy cohort (N=731) estimated using multivariable Cox regression adjusting for age, gender, race/ethnicity, smoking status, cancer histology, stage at initial diagnosis, and PD-L1 score. Performance status (PS) is categorized as good (0-1) or poor (2 or greater) based on clinical notes at time of treatment initiation. HR shown for (A) all patients in the first six months after treatment initiation and (B) among patients who survive six months or longer. **Square** symbols indicate the estimates of hazard ratio (HR). **Error bars** indicate the 95% confidence interval (CI).


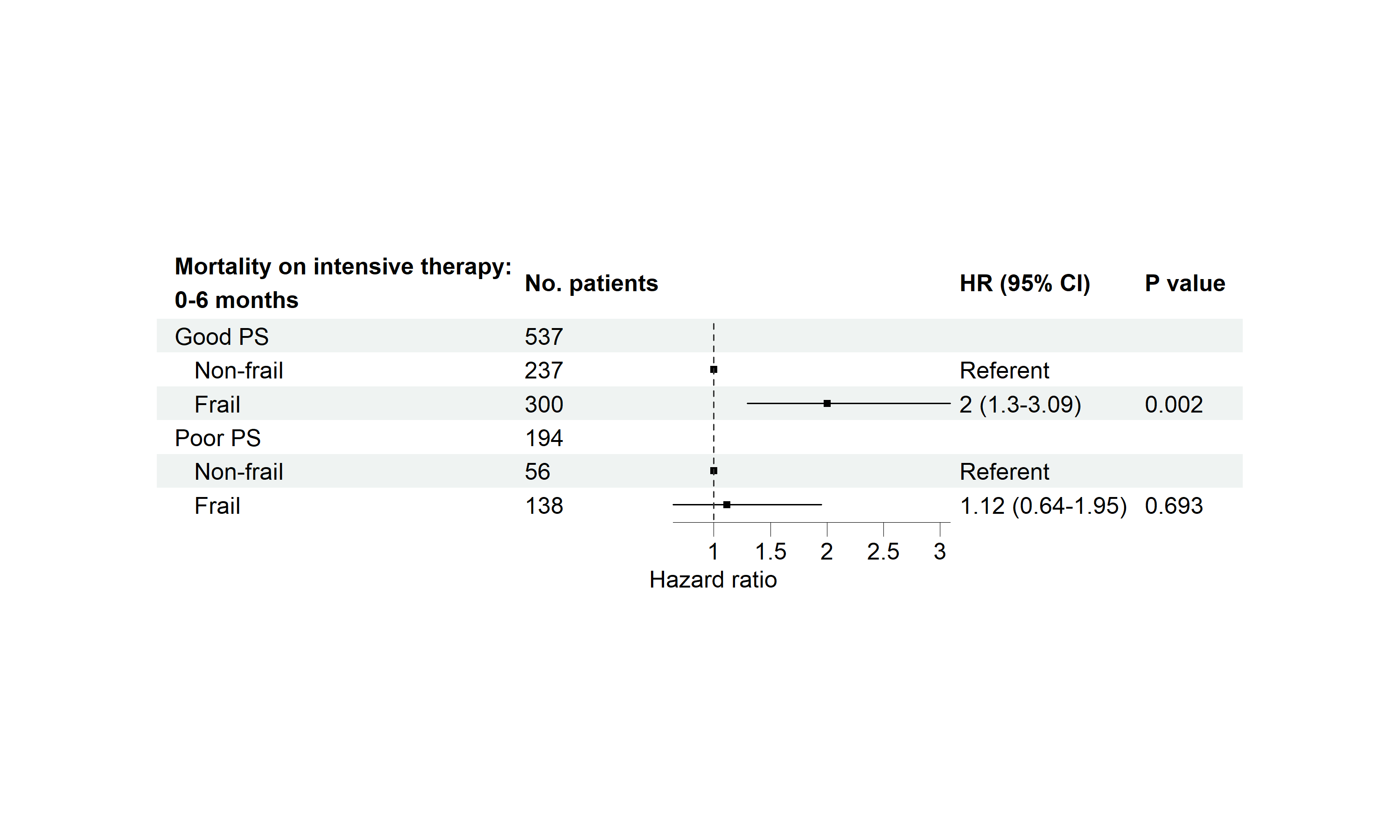

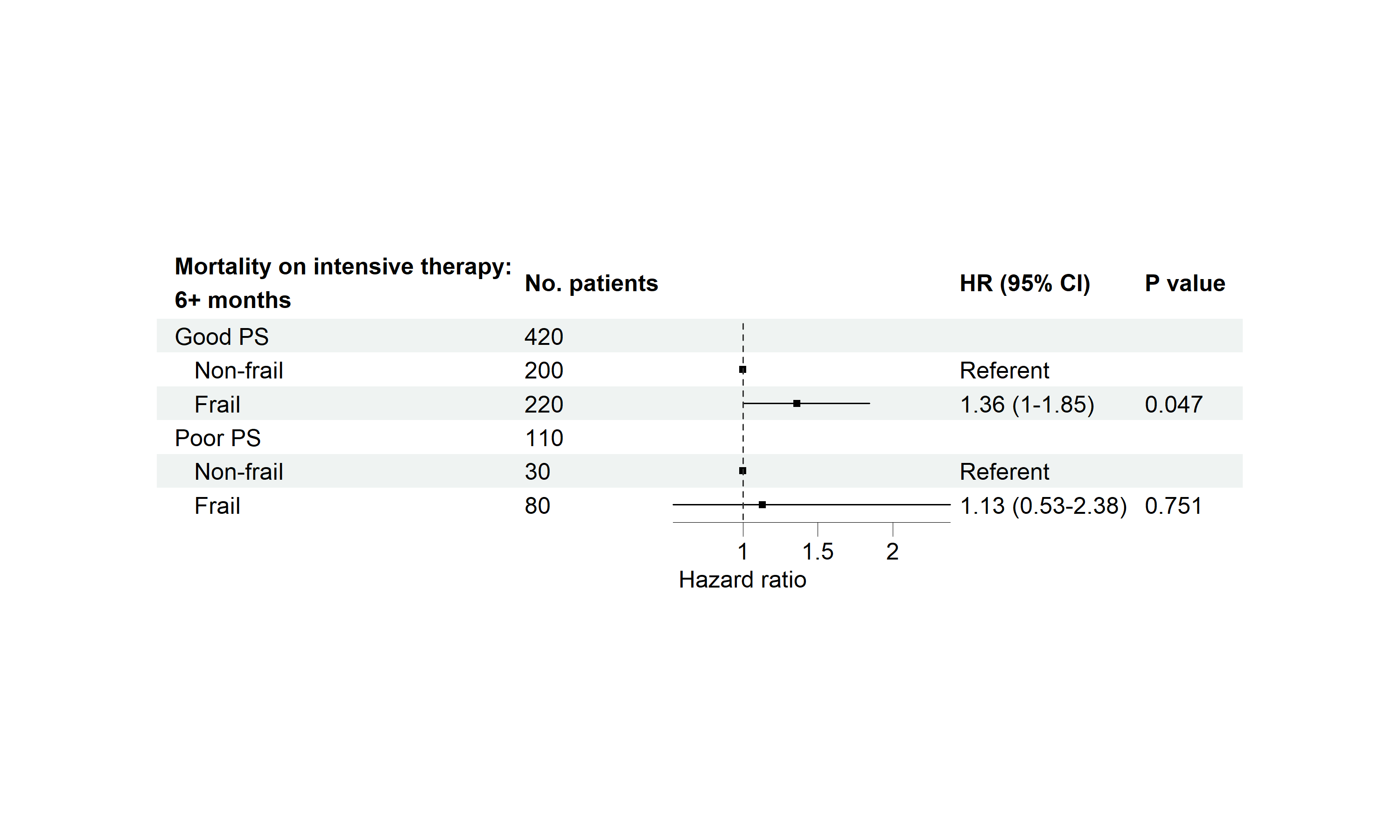


(A)

(B)

**Supplemental figure 5. Kaplan-Meier curves and association of frailty with overall survival on intensive therapy across PD-L1 expression levels.** Overall survival in the intensive therapy cohort (N=731) from the time of intensive therapy initiation. Intensive therapy is defined as first-line immune checkpoint inhibitor therapy with concurrent receipt of platinum-doublet chemotherapy and/or dual checkpoint blockade. Performance status (PS) is categorized as (A) good (0-1) or (B) poor (2 or greater) based on clinical notes at time of treatment initiation. Kaplan-Meier curves are shown separated by PD-L1 expression level: (A) negative (0%), (B) low (1-49%), and (C) high (≥50%). Forest plot of hazard ratio (HR) estimated using multivariable Cox regression adjusting for gender, race/ethnicity, smoking status, cancer histology, and stage at initial diagnosis. HRs shown for PD-L1: (D) negative, (E) low and (F) high. **Square** symbols indicate the estimates of HR. **Error bars** indicate the 95% confidence interval (CI).


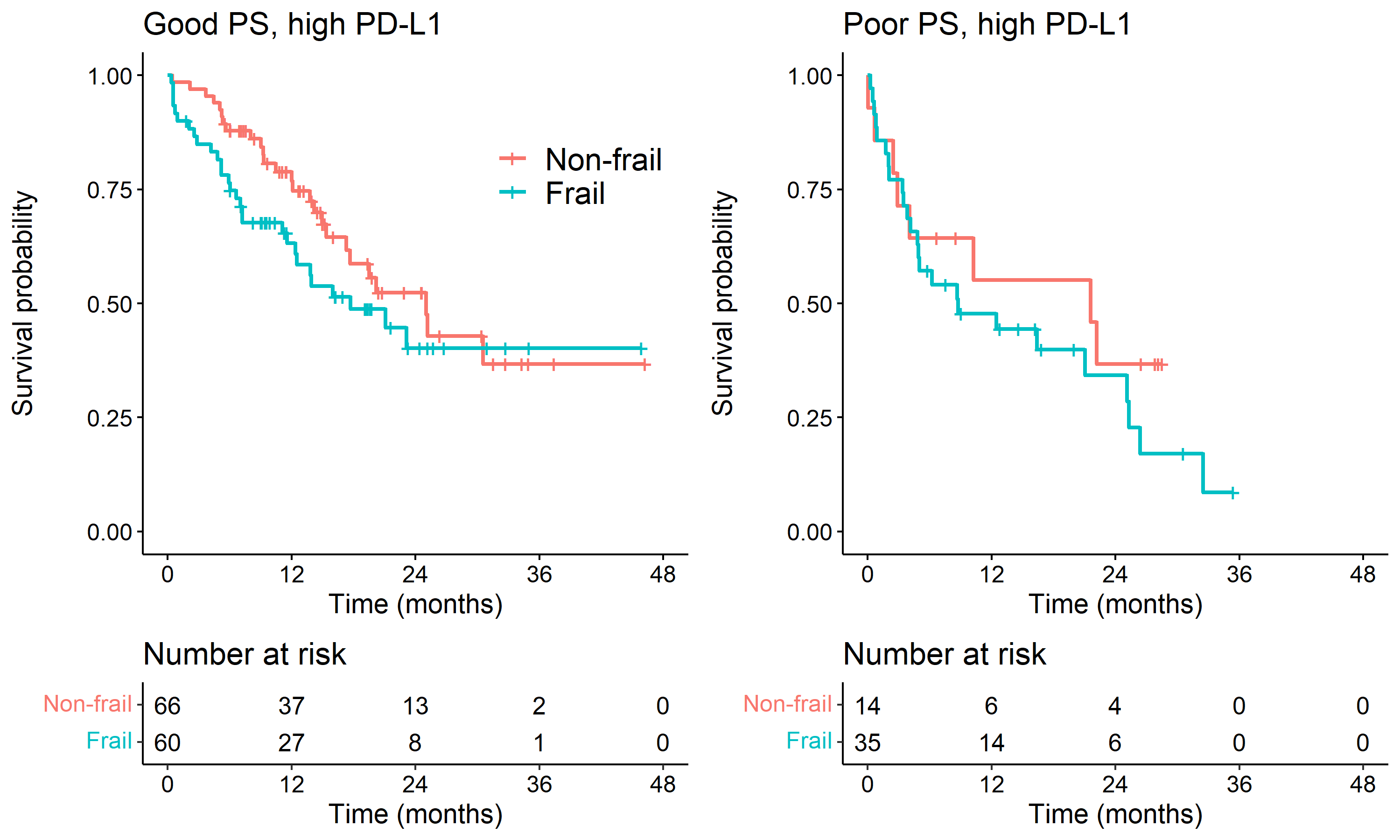

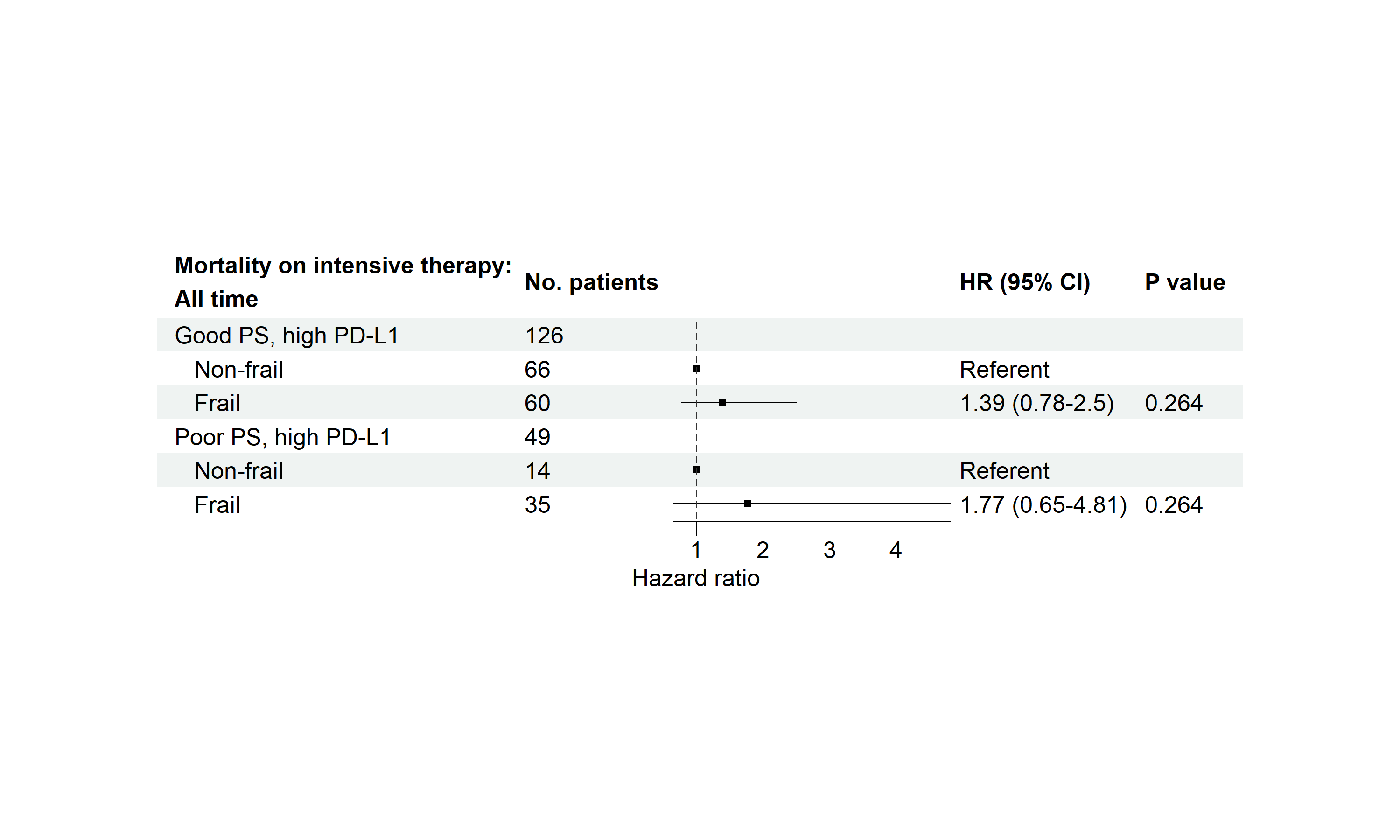


(A)

(D)


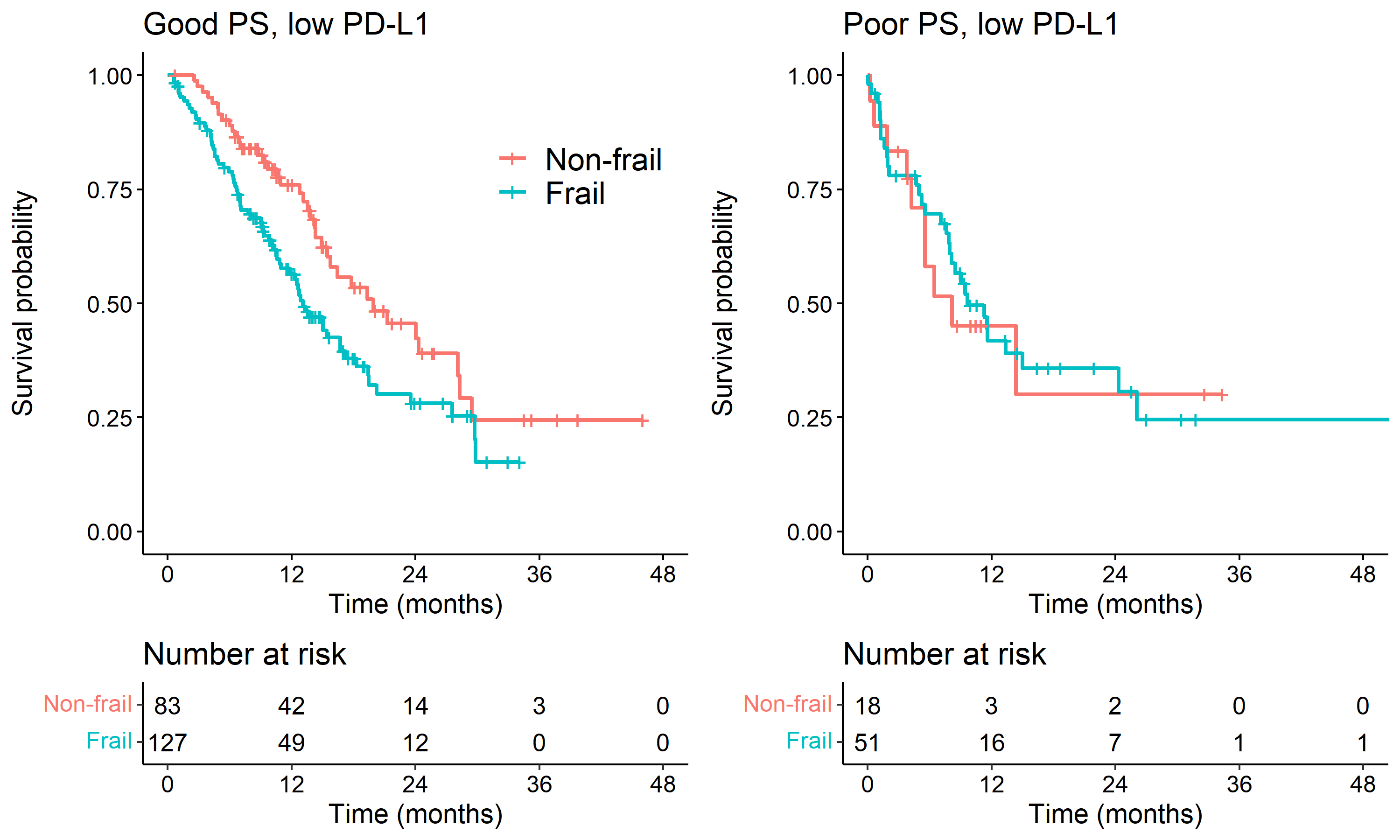

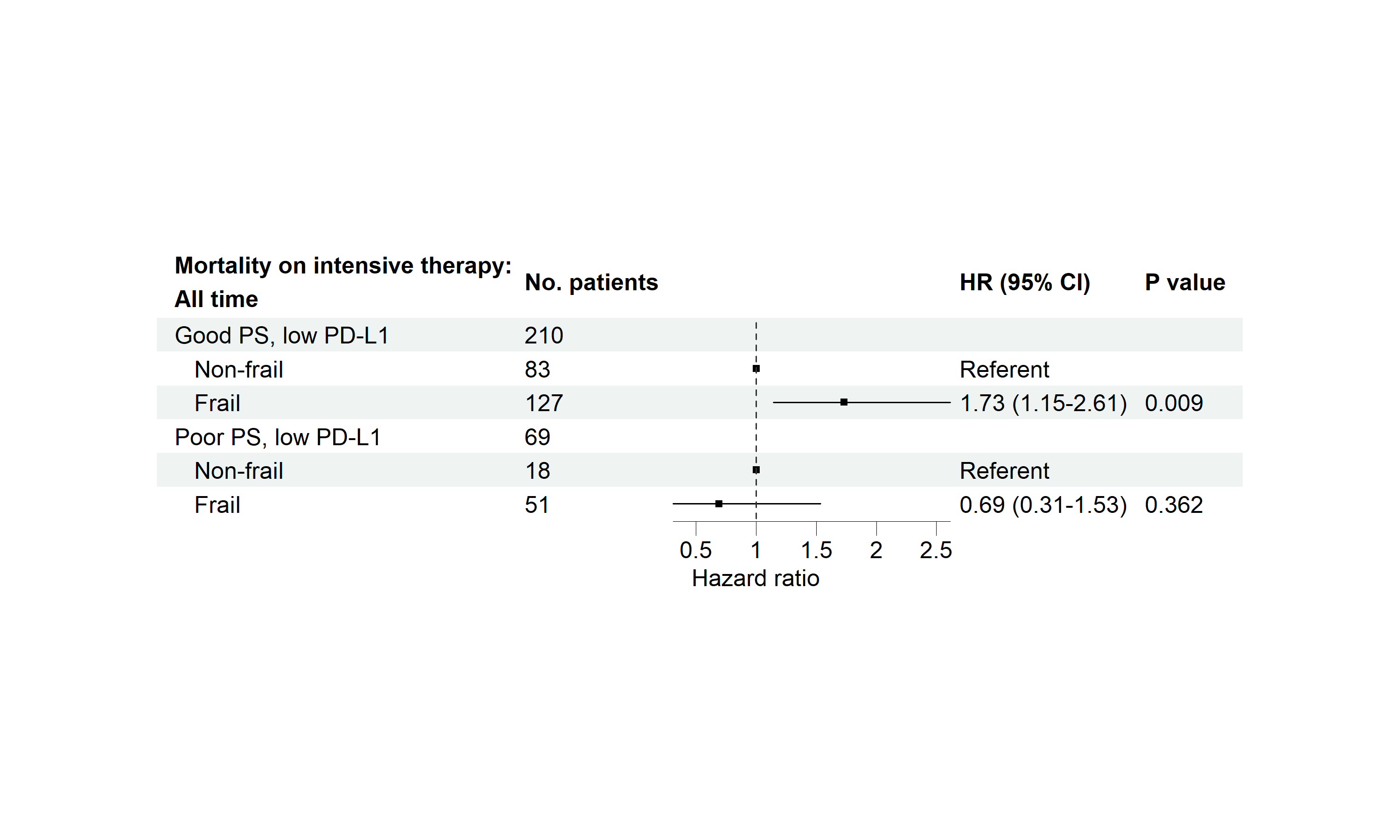


(B)

(E)


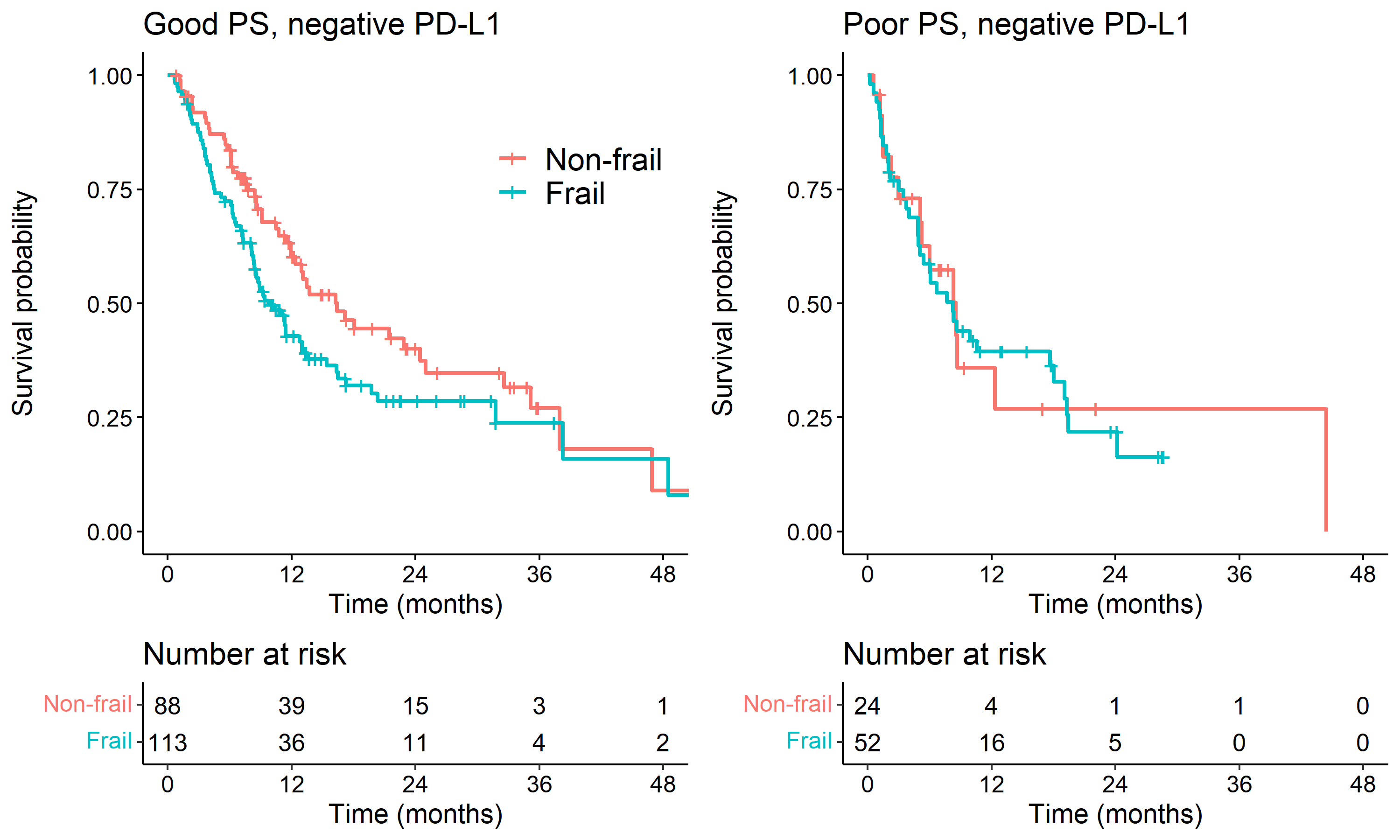

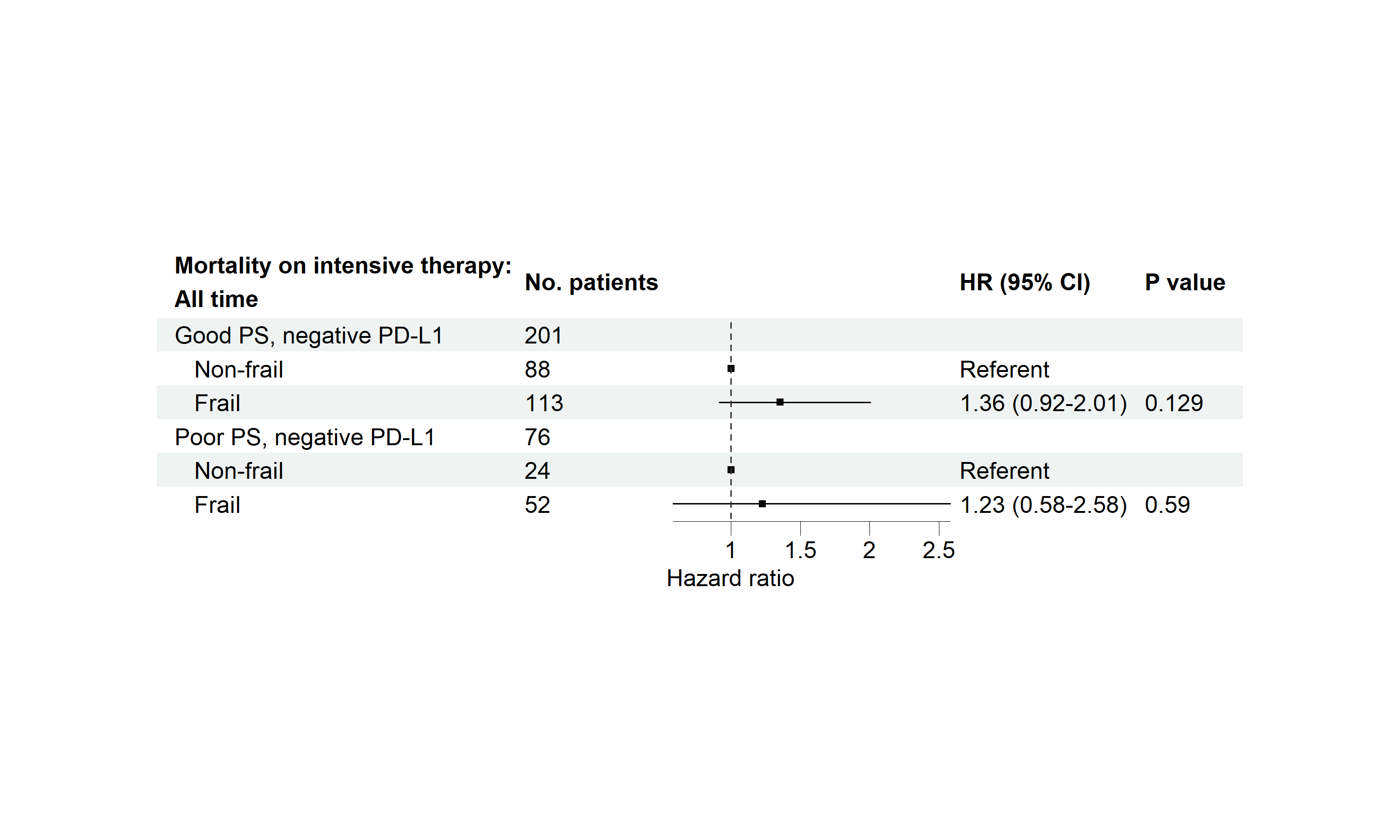


(C)

(F)

**Supplemental figure 6. Stratified analysis by performance status: association of frailty with overall survival on ICI monotherapy.** Overall survival in the ICI monotherapy cohort (N=816) from the time of ICI monotherapy initiation. Performance status (PS) is categorized as good (0-1) or poor (2 or greater) based on clinical notes at time of treatment initiation. Shown are Kaplan-Meier curves separated by frailty in patients with (A) good PS or (B) poor PS. (C) Forest plot of hazard ratio (HR) of overall survival estimated using multivariable Cox regression adjusting for age, gender, race/ethnicity, smoking status, cancer histology, stage at initial diagnosis, and PD-L1 score. **Square** symbols indicate the estimates of HR. **Error bars** indicate the 95% confidence interval (CI).


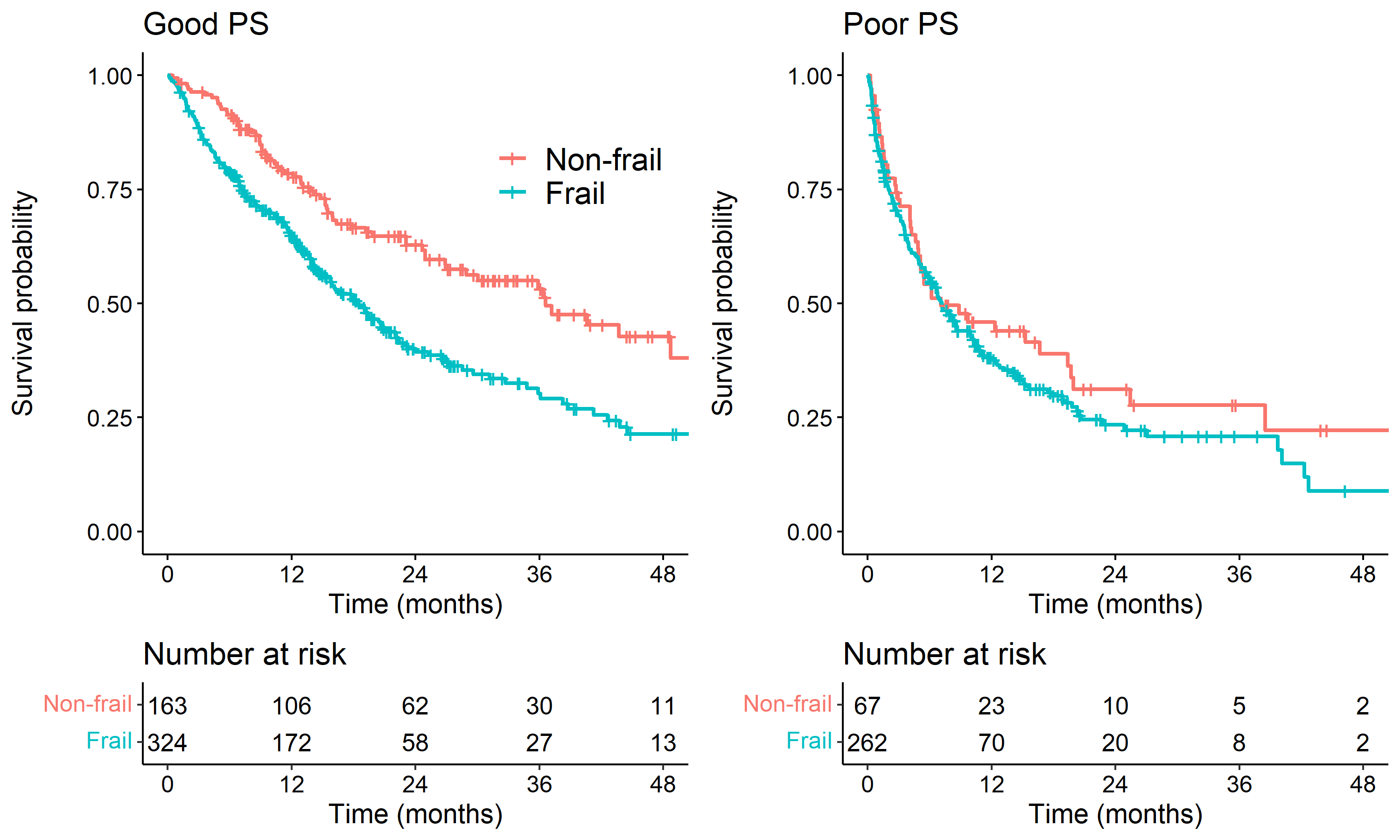

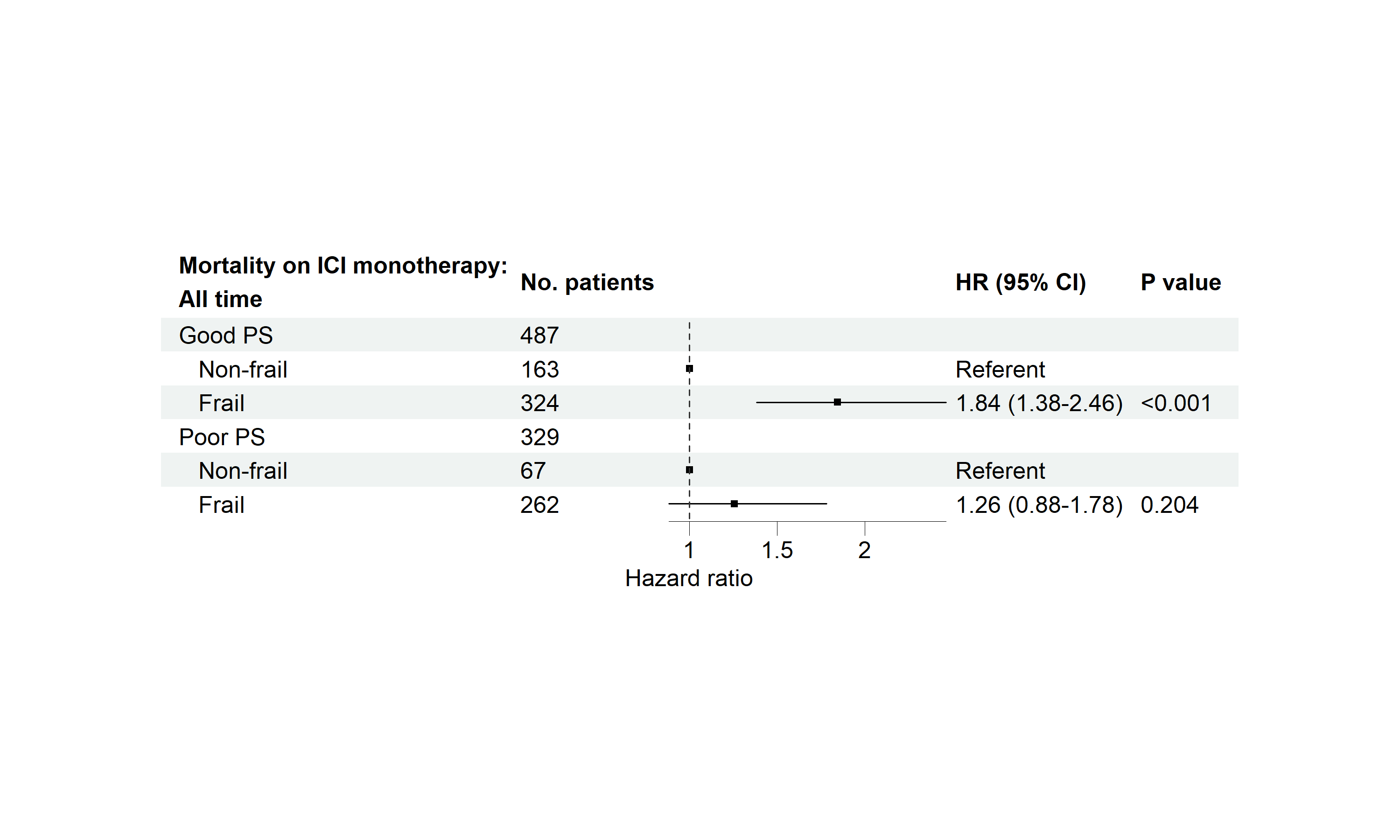


(A)

(B)

(C)

**Supplemental figure 7. Kaplan-Meier curves for overall survival on intensive therapy among patients aged <65y vs. 65+.** Overall survival in the intensive therapy cohort (N=731) from the time of intensive therapy initiation. Intensive therapy is defined as first-line immune checkpoint inhibitor therapy with concurrent receipt of platinum-doublet chemotherapy and/or dual checkpoint blockade. Performance status (PS) is categorized as (A) good (0-1) or (B) poor (2 or greater) based on clinical notes at time of treatment initiation. Age is separated into <65 years old versus 65 years or older based on age at treatment initiation.


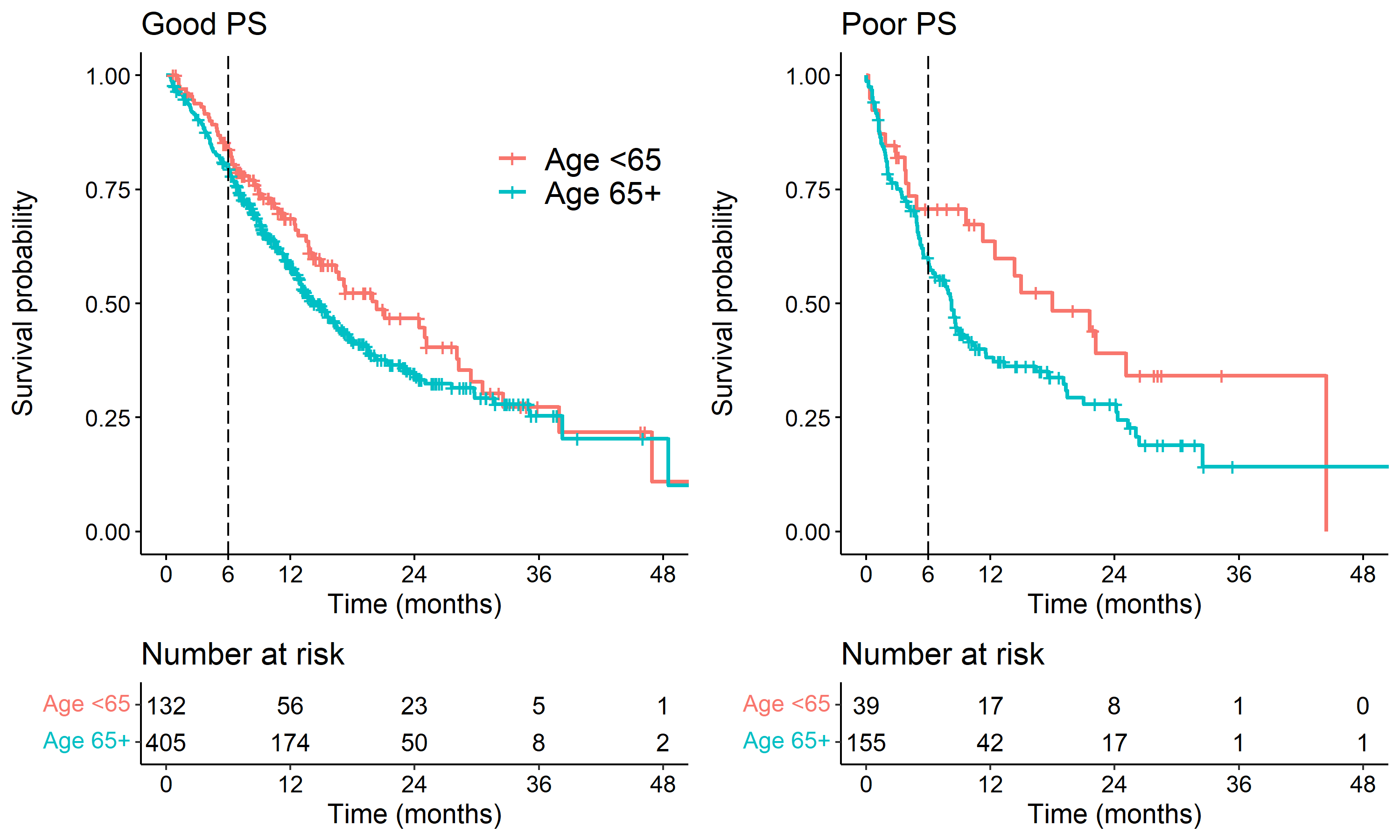


(A)

(B)

**Supplemental figure 8. Association of age with overall survival on intensive therapy when stratified by performance status.** Forest plot of hazard ratio (HR) of overall survival in the intensive therapy cohort (N=731) estimated using multivariable Cox regression adjusting for frailty, gender, race/ethnicity, smoking status, cancer histology, stage at initial diagnosis, and PD-L1 score. HR shown for (A) all patients, (B) all patients in the first six months after treatment initiation and (C) among patients who survive six months or longer. **Square** symbols indicate the estimates of HR. **Error bars** indicate the 95% confidence interval (CI).


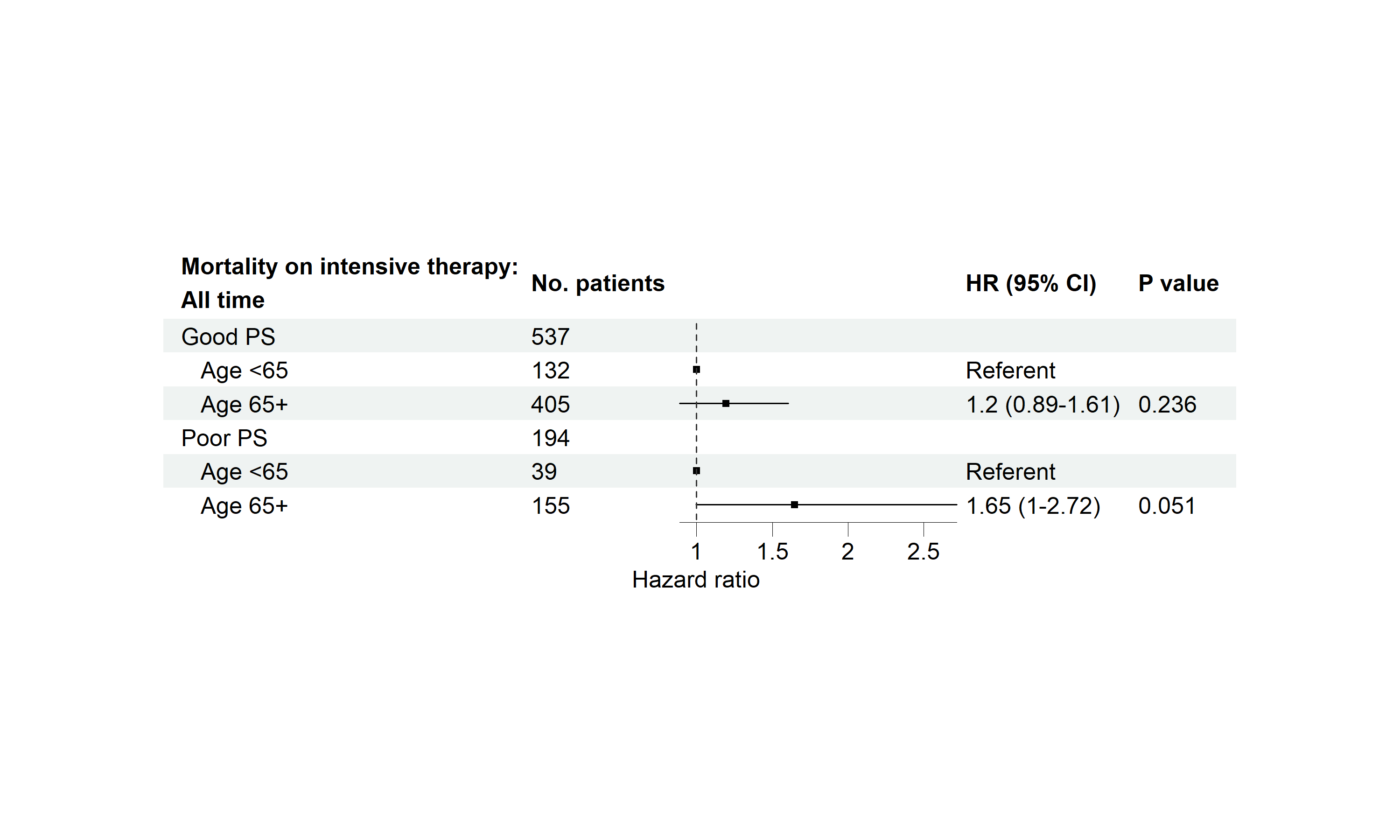

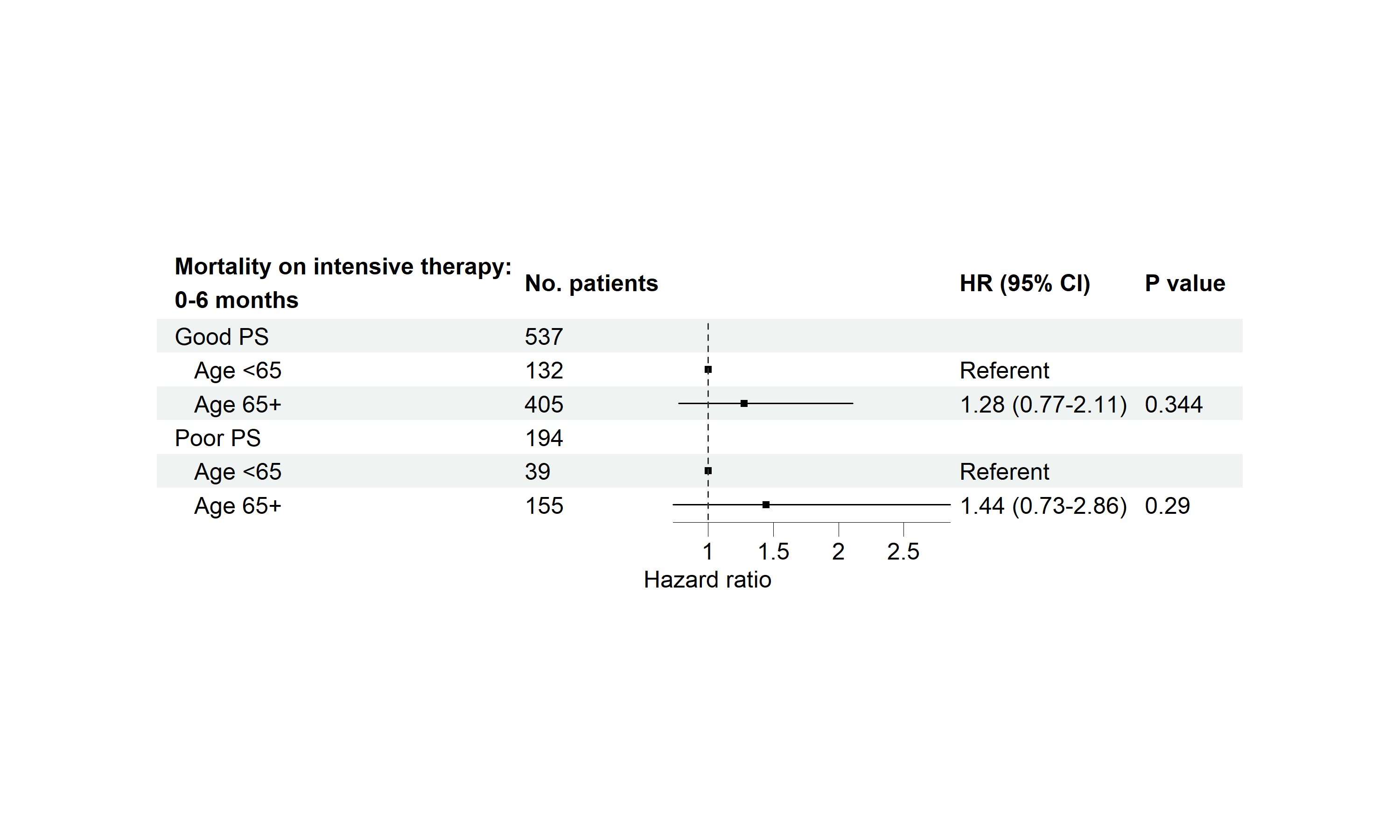

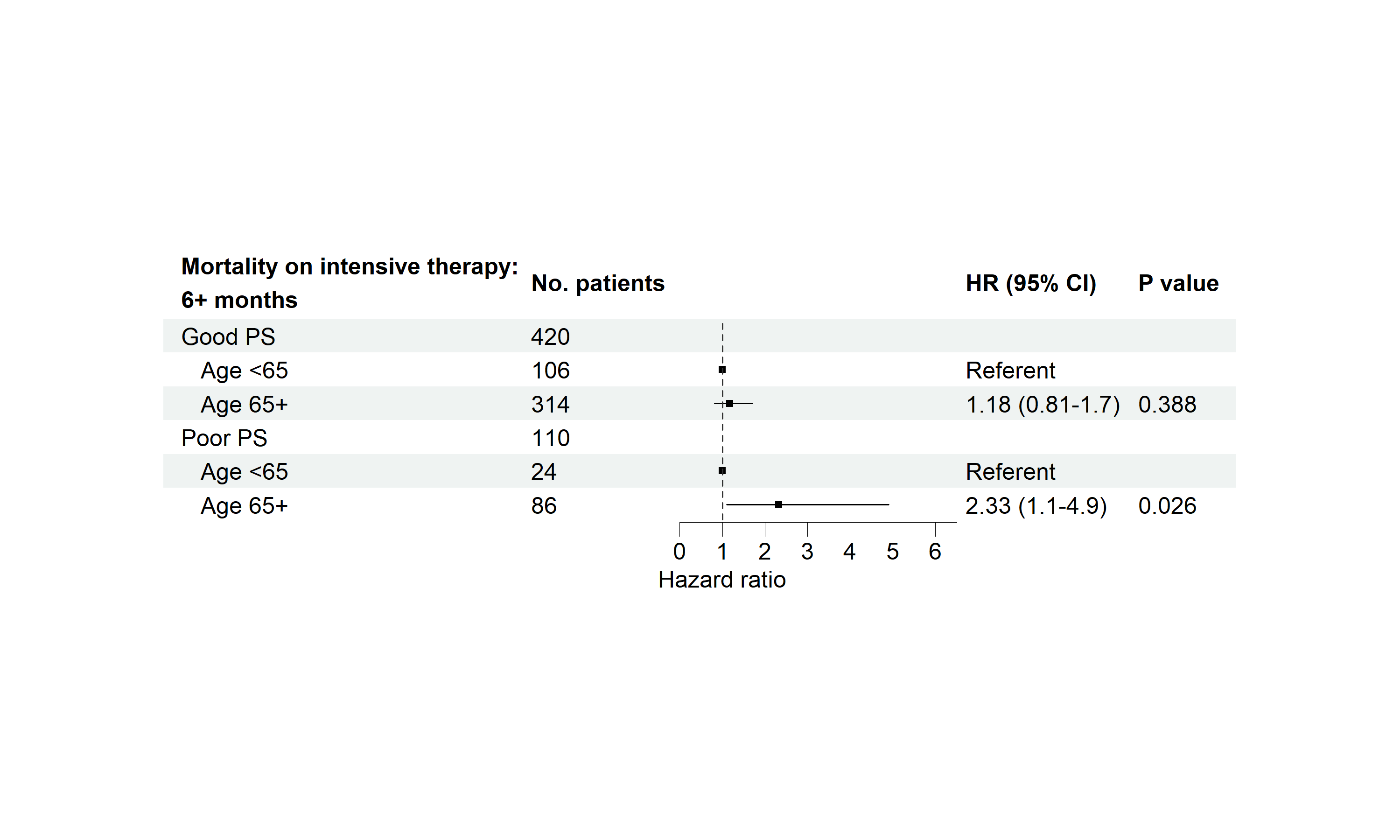


(A)

(B)

(C)
